# Naphthalene diimide bis-guanidinio-carbonyl-pyrrole as a pH-switchable threading DNA intercalator

**DOI:** 10.3762/bjoc.16.185

**Published:** 2020-09-08

**Authors:** Poulami Jana, Filip Šupljika, Carsten Schmuck, Ivo Piantanida

**Affiliations:** 1 Institute for Organic Chemistry, University of Duisburg-Essen, Universitässtrasse 7, 45141 Essen, Germany; 2 Integrated Science Education & Research Centre, Siksha-Bhavana, Visva-Bharati, Santiniketan-731235, India; 3 Division of Organic Chemistry and Biochemistry, Ruđer Bošković Institute, P. O. Box 180, HR-10002 Zagreb, Croatia,; 4 Laboratory for Physical Chemistry and Corrosion, Department of Chemistry and Biochemistry, Faculty of Food Technology and Biotechnology, University of Zagreb, Croatia

**Keywords:** AFM, circular dichroism, DNA/RNA recognition, fluorescence, guanidinio-carbonyl-pyrrole, naphthalene diimide

## Abstract

A novel naphthalene diimde analogue (NDI) equipped at the imide positions with two guanidinio-carbonyl-pyrrole (GCP) pendant arms interacted significantly stronger with ds-DNA at pH 5 than at pH 7, due to reversible protonation of the GCP arms. This was consequence of a pH-switchable threading intercalation into ds-DNAs only at pH 5, while at neutral conditions (pH 7) NDI-GCP_2_ switched to the DNA minor groove binding. Intriguingly, NDI-GCP_2_ was at both pH values studied bound to the ds-RNA major groove, still showing a higher affinity and thermal denaturation effect at pH 5 due to GCP protonation. At excess over the DNA/RNA conjugate NDI-GCP_2_ showed also aggregation along the ds-polynucleotide and AFM and DLS demonstrated that NDI-GCP_2_ has pronounced ds-DNA condensation ability.

## Introduction

The small molecules non-covalently binding to DNA or RNA are essential for life as we know it, and therefore it was not surprising that a huge number of synthetic small molecules has been prepared and studied for broad biochemical and biomedical applications [1–2]. Among the most studied small molecules were dyes used for the DNA/RNA labelling or even monitoring or influencing DNA/RNA function [3–4]. However, due to the complexity of DNA-coded or RNA-coded processes, also including epigenetics, there is permanent need for novel dyes, differing in selectivity, colour, or method of monitoring [5–6]. Among many strategies to achieve novel dye properties, one is based on combining two or more DNA/RNA binding modes in one small molecule (dye), thus building elaborate constructs which are able to recognise and report between slightly different DNA/RNA structures [2,7]. For the recognition monitoring, fluorescence is still the most popular method [8–11]. However, many new techniques or established ones with increased sensitivity are constantly improved or developed to report complementary to fluorescence. For instance, circular dichroism (CD) spectropolarimetry, highly sensitive to chiral properties of the DNA or RNA helical structures [12–13], could also take advantage of induced CD spectrum (ICD) in the visible spectrum range of small achiral dyes, which they show only upon binding to DNA/RNA [14]. Moreover, with recent advances in fluorescence emission-based polarisation spectroscopy methods (FDCD, CPL), the sensitivity of response is approaching the classical fluorimetric probes [14].

Our systematic work on aryl-guanidiniocarbonyl-pyrrole (GCP) derivatives characterised the GCP moiety as very useful building block in the design of new small molecules targeting DNA/RNA. The GCP moiety by itself does not interact with DNA/RNA but, if combined with oligomers [15–16] or attached to the aryl moiety containing at least two aromatic rings [17], it will ensure efficient DNA/RNA binding. Moreover, due to the weakly acidic p*K*_a_ = 6 of GCP, interactions and consequently recognition of various DNA/RNA sequences could be reversibly changed by adjusting the pH of the solution. The fine structural tuning of such aryl-GCP conjugates allowed us to prepare a wide variety of small molecules with different DNA or RNA sequence selectivity, many of them acting as multipurpose probes – binding to several different DNA or RNA structures but for each of them giving a different spectrophotometric response [17–22].

Until now we have studied aryl systems which behaved as ds-DNA/RNA intercalators, DNA/RNA groove binders, or nucleobase derivatives. In this work we focused our attention to a less common DNA/RNA binding motif: threading intercalation. This sterically very demanding binding mode is characterised by a central large aromatic moiety, in this case the well-known naphthalene diimide (NDI), equipped at both sides of the long axis with large substituents, which have to thread through DNA double helix and are therefore positioned in the minor and major groove, respectively [23–25].

**Scheme 1:**
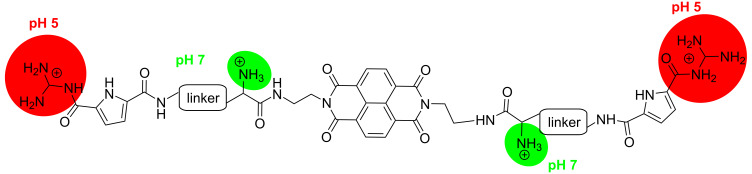
DNA-targeting roles of the structural components incorporated in the design of the novel small molecule. The NDI chromophore (DNA threading intercalator or possible groove binder) bearing two positive charges at adjacent amino groups (pH 7, green) and decorated by two GCP arms protonated at pH 5 (red; pH-switchable DNA groove binders).

Since GCP units always interact within DNA/RNA grooves, thus our particular interest in threading intercalation is the positioning of side chains simultaneously in both, the minor and major groove; in that way allowing two GCP units different recognition environment and therefore eventually better sensing of ds-polynucleotide secondary structures. However, if for some particular ds-DNA or ds-RNA threading intercalation is hampered, such novel NDI-(GCP)_2_ conjugate (**4**; Scheme 1) will most likely bind within one of the DNA/RNA grooves, as in previously described aryl-GCP conjugates [16–22].

Further, the designed novel naphthalene diimide (NDI) analogue **4** (Scheme 1) is characterized by four pH-dependent positive charges, two of them present at neutral conditions (pH 7, amino groups close to the NDI unit, green) and two protonated only at weakly acidic conditions (pH 5; acyl guanidinium unit in the GCP moiety, red) positioned at the ends of the pendant arms. Thus, pH control of a number of positive charges can additionally and reversibly control interactions with DNA/RNA.

To study the interactions of **4** with DNA/RNA, we opted for long (>100 base pairs) polynucleotides, to ensure a large number of identical binding sites along one polymer (Table S2 in Supporting Information File 1). Thus, we used calf thymus (ct)-DNA as a typical B-helix structure with a balanced ratio of GC-(48%) and AT-(52%) base pairs, as well as synthetic alternating polynucleotide poly(dAdT)_2_ with also B-helix structure but with a fully available minor groove for small molecule binding. As alternative we used poly(dGdC)_2_ differing significantly in the secondary structure as well as in the availability of the minor groove for small molecule binding (the guanine amino group sterically hinders deep molecule penetration). Homopolynucleotide poly(dA)–poly(dT) is characterised by a peculiar twisted C-DNA structure with a minor groove only half-width of the minor groove of the alternating analogue poly(dAdT)_2_ (Table S2, Supporting Information File 1). For comparison between double-stranded (ds) DNA and ds-RNA, poly(rA)–poly(rU) was chosen as an A-helical structure having a major groove convenient for binding of bulky small molecules.

## Results and Discussion

### Synthesis

**Scheme 2:**
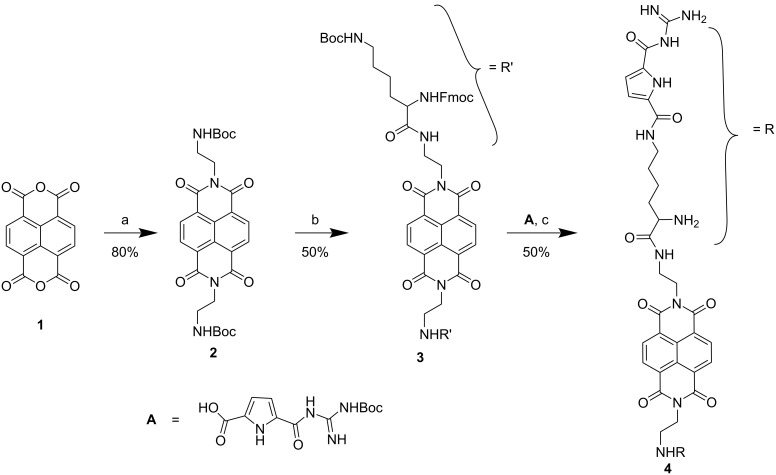
Synthesis of compound **4**. Conditions: a) mono-*N*-Boc-ethylenediamine, DMF, 90 °C; b) i) trifluoroacetic acid (TFA)/CH_2_Cl_2_ 1:1, ii) Fmoc-Lys(Boc)-OH, DIPEA, HCTU; c) i) TFA/CH_2_Cl_2_ 1:1, ii) GCP, **A**, DIPEA, HCTU, iii) 20% piperidine in DMF, iv) TFA/CH_2_Cl_2_ 1:1.

The synthesis of compound **4** was performed as outlined in Scheme 2 and the detailed description is given in the Experimental section.

### Physicochemical and spectroscopic properties of aqueous solutions of **4**

Compound **4** is well-soluble in water (10^−3^ M range), whereby at neutral conditions (pH 7) compound **4** bears two positive charges, while at pH 5 both GCP moieties are also protonated, yielding **4** with four positive charges [18]. The concentration dependence of **4** according to its UV–vis spectrum at both, pH 5 and 7, was linear up to 2 × 10^−5^ M (Supporting Information File 1), supporting the presence of single, non-aggregated **4** molecules in water. The absorption maxima and corresponding molar extinction coefficients (ε) are given in Table S1 (Supporting Information File 1). Heating of the aqueous solution of **4** up to 90 °C did not yield any significant changes in its UV–vis spectrum, suggesting that the chromophores are not involved in intra- or intermolecular stacking interactions. The excellent reproducibility of the UV–vis spectrum upon cooling back to room temperature (Figure S2, Supporting Information File 1) verified the chemical stability of the compound. The aqueous solution of the studied compound **4** was non-fluorescent at experimental conditions and addition. Since NDI dyes are known to emit fluorescence, we also tried fluorimetric titrations with the hope that the initially non-emissive compound **4** becomes fluorescent upon binding to DNA (for instance cyanine dyes and many other behave that way). However, we did not observe any emission, neither for single dye binding to DNA, nor in crowding conditions (excess of dye over DNA binding sites) at which eventually fluorescent excimers could be formed.

### Interactions with DNA/RNA

Because of significant differences in the protonation states of compound **4**, studies were performed at pH 7 (**4** net charge 2^+^) and pH 5 (**4** net charge 4^+^). In thermal denaturation experiments at pH 7 compound **4** did not significantly stabilise ds-DNA or ds-RNA (Figures S10 and S11 in Supporting Information File 1), while at pH 5 all ds-polynucleotides were strongly stabilised (Table 1, and Figures S12–S15 in Supporting Information File 1). The latter efficient stabilisation could be attributed to the protonation of two GCP units at weakly acidic conditions [17] and subsequently additional interactions with the ds-polynucleotide.

**Table 1:** Thermal stabilisation (∆*T*_m_^a^/°C) of ds-DNA and ds-RNA upon the addition of compound **4** at various ratios *r*^b^ at pH 5 (buffer sodium cacodylate, *I* = 0.05 M).

*r*^b^	ct-DNA	poly(dAdT)_2_	poly(dA)–poly(dT)	poly(rA)–poly(rU)

0.05	3.1	–	–	–
0.1	9.1	10.0	10.5	1.1/6.6^c^
0.2	10.9	–	–	6.8
0.3	d	12.1	10.1	d

^a^Error in ∆*T*_m_ ± 0.5 °C. ^b^*r* = [**4**]/[polynucleotide]. ^c^Biphasic thermal denaturation transition due to partial saturation of ds-RNA. ^d^Precipitation.

For all ds-polynucleotides the thermal stabilisation effect (positive ∆*T*_m_ values) reached saturation within *r*_[_**_4_**_]/[polynucleotide]_* ≈* 0.2, suggesting that at this ratio all dominant binding sites of compound **4** along DNA/RNA were occupied. Moreover, the lack of stabilisation at pH 7 excluded the NDI-core intercalation into ds-polynucleotide.

**Figure 1:**
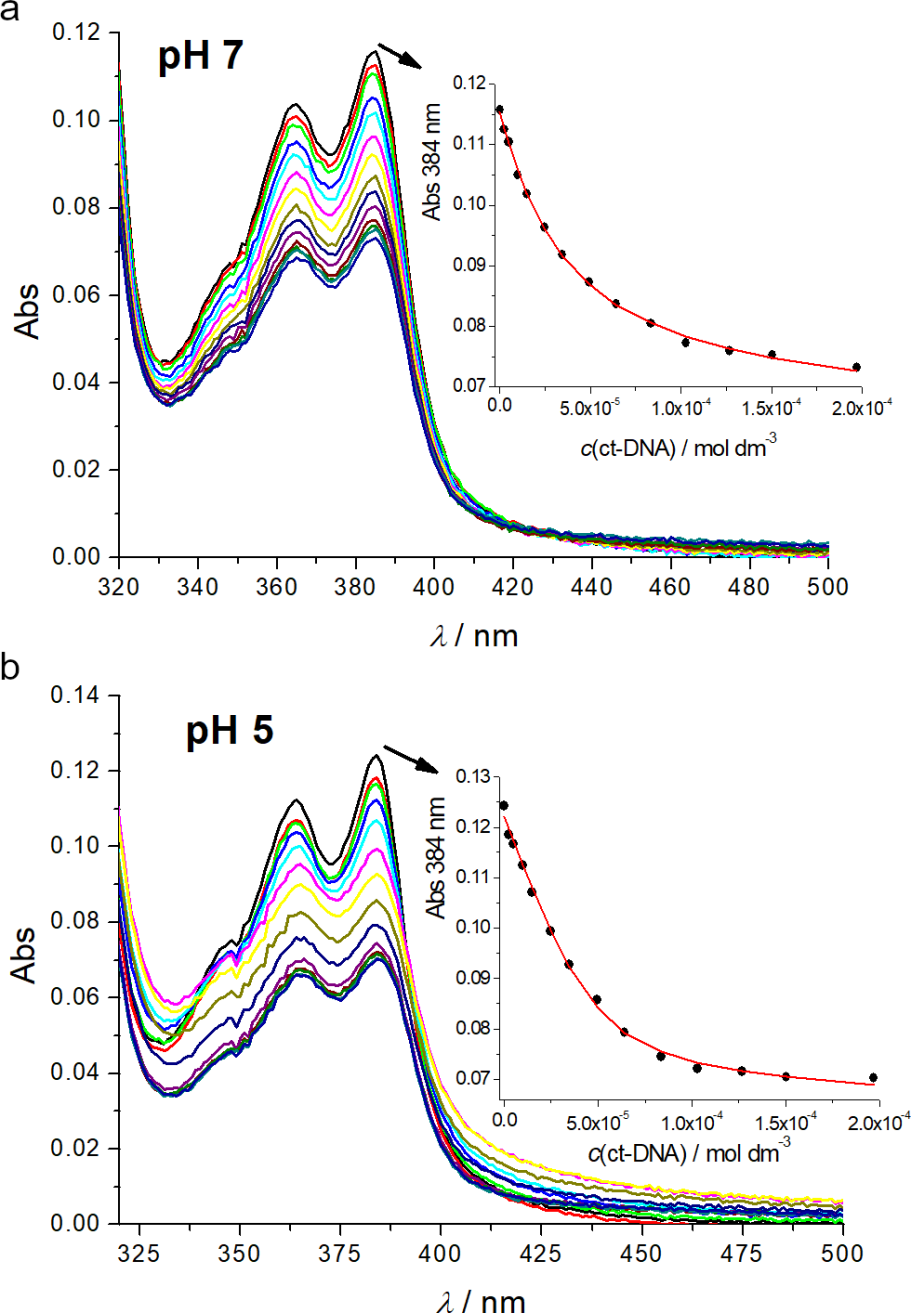
UV–vis titrations of compound **4** (*c* = 1.0 × 10^−6^ M), with ct-DNA at pH 7.0 (a) and at pH 5 (b). Insets: dependence of Abs (384 nm) on *c*(ct-DNA), line – non-linear fit to Scatchard equation [28]. Measurement done in Na cacodylate buffer, *I* = 0.05 M.

Further, we performed UV–vis titrations of **4** and addition of any of the studied ds-polynucleotides revealed strong hypochromic effect on the dye’s spectrum at λ > 300 nm, irrespectively of pH (Figure 1 and Figures S3–S9 in Supporting Information File 1). Since a suspected threading intercalation of NDI usually requires longer incubation times [23,26–27], the time required for reaching equilibrium was checked by repeatedly collecting UV–vis spectra of compound **4** upon the additions of DNA or RNA aliquots to the dye solution in 10 s intervals. The results showed that an incubation period of 180 s proved to be sufficient for reaching thermodynamic equilibrium. It should be stressed that all changes in the 330–400 nm range could be attributed dominantly to the NDI chromophore, since absorbance of GCPs in this range is negligible. Absorbance changes of large aromatic chromophores upon DNA or RNA addition can happen for many reasons, the most common ones being solvatochromic effects or aromatic stacking interactions. In this particular case, the NDI chromophore in threading intercalation has to be stacked with two base pairs, causing typical hypochromic and bathochromic effects. However, the positioning of the NDI chromophore in the DNA/RNA grooves aside expulsion of water solvating the NDI, also allows for aromatic interactions, either edge-to-face with base pairs or even with another NDI molecule (aggregation within groove). Both events strongly affect the UV–vis spectrum of the NDI chromophore. In any case, strong hypochromic and bathochromic changes are the consequence of non-covalent binding of compound **4** to DNA or RNA, thus allowing accurate processing of the titration data by non-linear fitting to the Scatchard equation [28], to determine the binding constants and Scatchard ratio *n*_[bound _**_4_**_]/[polynucleotide]_ (Table 2).

**Table 2:** Binding constants (log *K*_a_)^a^, ratios *n* = [**4**]/[polynucleotide] of **4** with ds-polynucleotides calculated from UV–vis titrations at pH 7 and pH 5 (buffer: sodium cacodylate, *I* = 0.05 M).

	ct-DNA	poly(dAdT)_2_	poly(dGdC)_2_	poly(dA)–poly(dT)	poly(rA)–poly(rU)

pH 7	4.8	nd	nd	nd	4.5
pH 5	5.8	5.8	7.2	6.4	6.0

^a^Processing of titration data by means of Scatchard equation [28] gave values of the ratio *n*_[bound _**_4_**_]/ [polynucleotide]_ = 0.2 –0.3; for easier comparison all log *K*_a_ values were re-calculated for fixed *n* = 0.2. Correlation coefficients were >0.99 for all calculated *K*_a_.

The detailed analysis of results (Table 2) revealed that at neutral conditions (pH 7) compound **4** showed at least one order of magnitude lower binding constant for ds-DNA and ds-RNA in comparison to the affinity at pH 5, agreeing well with the thermal denaturation results (Table 1), which can be attributed to the positively charged GCP units at acidic conditions. A comparison of compound’s **4** binding constants at pH 5 revealed a one order of magnitude higher affinity of **4** toward GC-DNA in comparison to other AT(U)-containing ds-polynucleotides. Such selectivity is in line with the number of typical intercalators, particularly those which have substituents protruding into either the major or minor grooves (or both) [29].

For a more detailed structural analysis of the complexes, CD spectroscopy was applied [12–13]. It should be noted that due to the distance between the asymmetric atom and NDI or GCP chromophores, the intrinsic CD spectrum of compound **4** at the 220–400 nm range is negligible. Further, for the analysis of the CD results we compared our data with those published for a close NDI-monomer analogue [26–27], whereby the strong positive ICD band at 270–280 nm was attributed to the intercalation of NDI into ds-DNA. Also, we showed previously that positioning of the GCP-unit into the DNA minor groove resulted in a strong positive ICD band at 300–320 nm [13,18–19].

**Figure 2:**
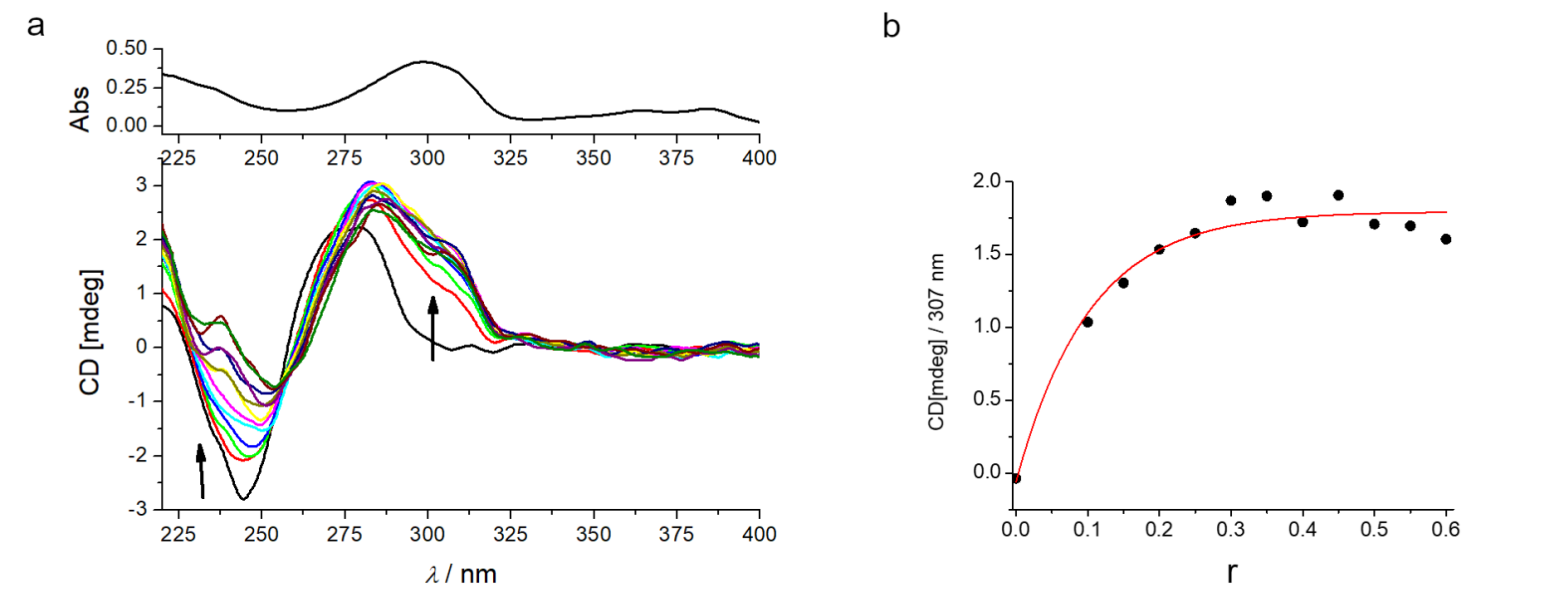
a) CD titration of ct-DNA (*c* = 3.0 × 10^−5^ M) with compound **4** at pH 7.0 (Na cacodylate buffer, *I* = 0.05 M). b) Dependence of the ICD intensity at 307 nm on the molar ratios *r*_[_**_4_**_]/[ctDNA]_; note saturation of binding sites at *r* = 0.2.

At pH 7 the CD spectrum of ct-DNA upon titration with compound **4** (Figure 2a) showed significant changes. The intensity of the negative DNA band at 245 nm decreased pointing to partial unwinding of the double helix, whereas the positive DNA band at 270–280 nm marginally increased. Most importantly, in the 300–330 nm range a strong ICD band appeared, agreeing well with the positioning of the GCP units within the minor groove [13,18–19]. Thus, all mentioned changes supported binding of the whole molecule of **4** in the DNA minor groove. The saturation of ICD band intensity (Figure 2b) at *r* = 0.2–0.3 nicely agreed with the obtained value of ratio *n*_[bound _**_4_**_]/[polynucleotide]_ from the Scatchard fitting results (Table 2). The absence of ICD bands within the naphthalene diimide absorption range (350–420 nm) suggested that the NDI transition dipole moment associated with the higher energy transition (NDI longer axis) was not well-oriented in respect to the DNA chiral axis [14].

**Figure 3:**
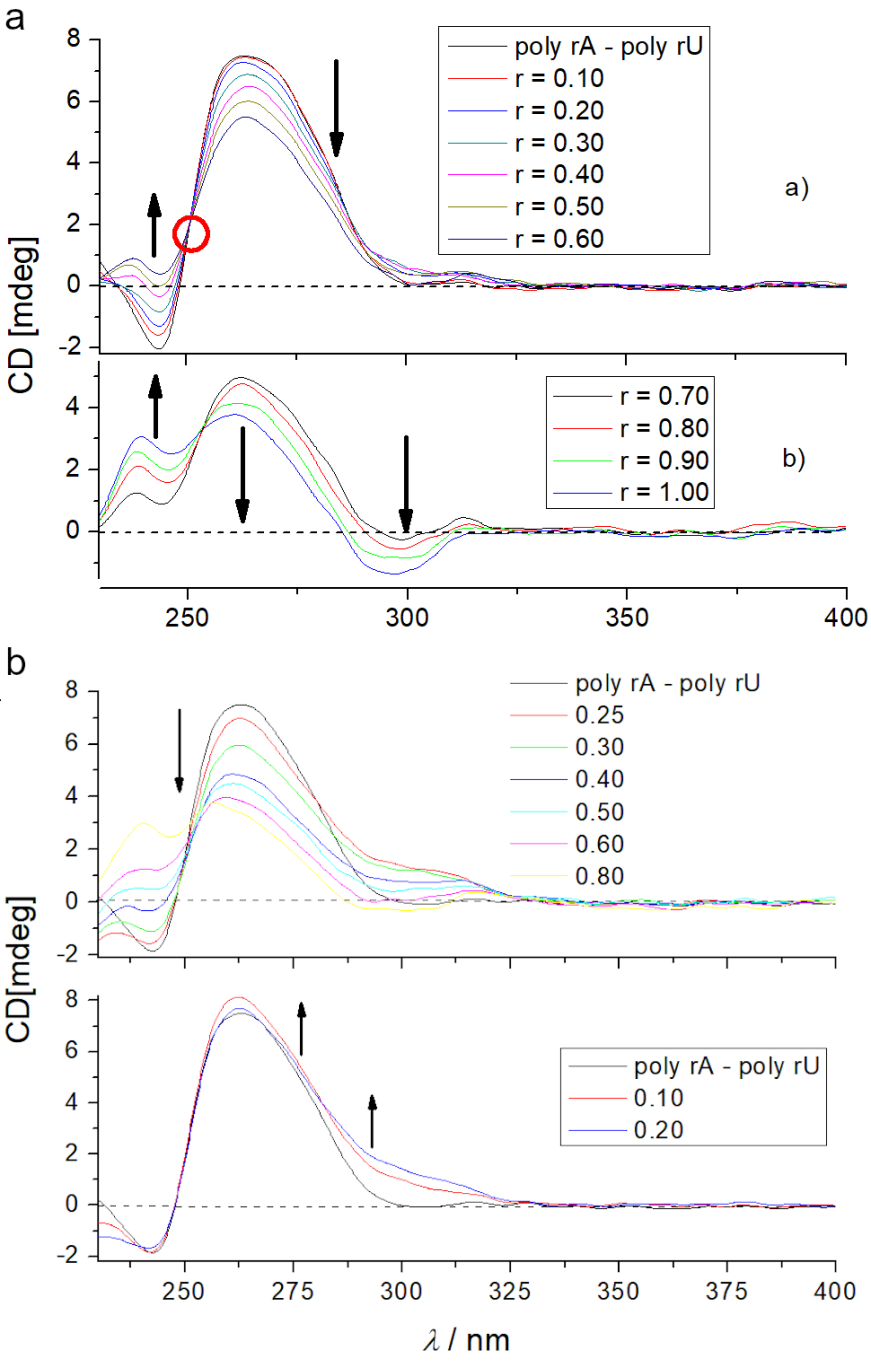
CD titration of poly(rA)–poly(rU) (*c* = 3.0 × 10^−5^ M) with compound **4** at a) pH 7.0 (Na cacodylate buffer, *I* = 0.05 M); top) ratio *r*_[_**_4_**_]/[RNA]_ = 0.1–0.6; bottom) ratio *r*_[_**_4_**_]_**_/_**_[RNA]_ = 0.7–1.0. The red circle denotes isoelliptic point at 250 nm. b) pH 7.0 (Na cacodylate buffer, *I* = 0.05 M); top) ratio *r*_[_**_4_**_]/[RNA]_ = 0–0.2; bottom) ratio *r*_[_**_4_**_]_**_/_**_[RNA]_ = 0.25–0.8.

At variance to ds-DNA, the addition of compound **4** to ds-RNA at pH 7 (Figure 3a) resulted in significantly different changes of the CD spectrum, whereby at least two different binding events could be clearly distinguished. At excess of RNA over **4** (Figure 3a, top), the clear isoelliptic point at 250 nm supported a dominant binding event, at which each molecule of **4** binds at separate RNA binding sites, whereas at large compound **4** excess over RNA (ratios *r* > 0.6, Figure 3a, bottom) a new, negative ICD band at 300 nm appeared, suggesting a secondary binding event, most likely caused by aggregation of **4** within the RNA major groove [14]. Again, there was no ICD band characteristic for NDI intercalation [26–27]. At pH 5 the **4**/ds-RNA complex changes in the CD spectrum (Figure 3b) were somewhat different in respect to those observed at pH 7 (Figure 3a), but again showing no ICD bands in the naphthalene diimide absorption range characteristic for intercalation. Thus, the observed CD results supported binding of compound **4** within the RNA major groove, consisting of dominant mode at *r* < 0.2 and aggregate type of binding at *r* > 0.2.

**Figure 4:**
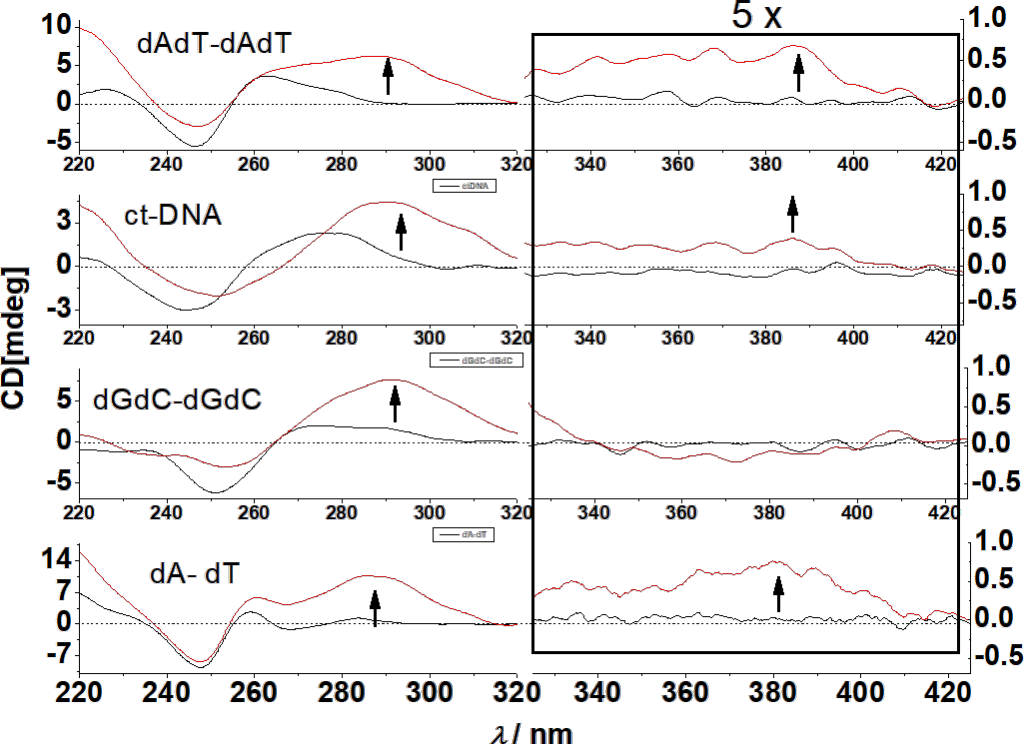
CD titration of ds-DNAs (*c* = 3.0 × 10^−5^ M) with **4** for ratio *r*_[_**_4_**_]/ [DNA]_ = 0.4. Done at pH 5.0 (Na cacodylate buffer, *I* = 0.05 M). Note that Y-scale in the 320–420 nm range was multiplied by 5.

At pH 5 the addition of **4** to ds-DNAs yielded significantly different CD spectra in comparison to pH 7. Namely, there was a considerable increase of CD bands at 270–280 nm range and additional weak positive ICD bands within the naphthalene diimide absorption range (350–420 nm) were observed but only for AT-containing DNA and not for GC-DNA (Figure 4).

Such ICD bands corroborated by strong thermal stabilisation (Table 1) and higher binding constants (Table 2) support an intercalative binding of the naphthalene diimide unit in AT-DNAs [26–27], whereby the positive sign of the ICD band (380 nm) supports a perpendicular orientation of the longer axis of the naphthalene diimide in respect to the longer axes of DNA base pairs [13–14], agreeing well with threading intercalation. The **4**/GC-DNA complex also showed a strong ICD band at 270–290 nm, typical for NDI intercalation [26–27], and the binding constant was exceptionally high. The absence of an ICD band at 350–420 nm suggested a different angle of the NDI longer axis in respect to the base pair longer axis, thus yielding an ICD band of negligible intensity [14].

Very importantly, the CD experiments at pH 5 clearly showed that at excess of **4** over the DNA/RNA dominant binding sites (*r* > 0.3) an additional binding mode of compound **4** is present, likely of lower affinity but nevertheless obvious by systematic deviation from isoelliptic point and changes of ICD bands in opposite direction (Figures 2–4, and Figures S16–S20 in Supporting Information File 1).

Hence, to characterise in more detail the thermodynamics of such multifaceted binding we performed isothermal titration calorimetry experiments (ITC), which allowed us to determine all thermodynamic components simultaneously in a single experiment (the equilibrium binding constant (*K*_a_), reaction Gibbs free energy of binding (Δ_r_*G*), reaction enthalpy (Δ_r_*H*), reaction entropy (Δ_r_*S*), and the stoichiometry (*n*) of the complex formed (see Experimental and Supporting Information File 1, Figures S21–S24) [30–32].

**Figure 5:**
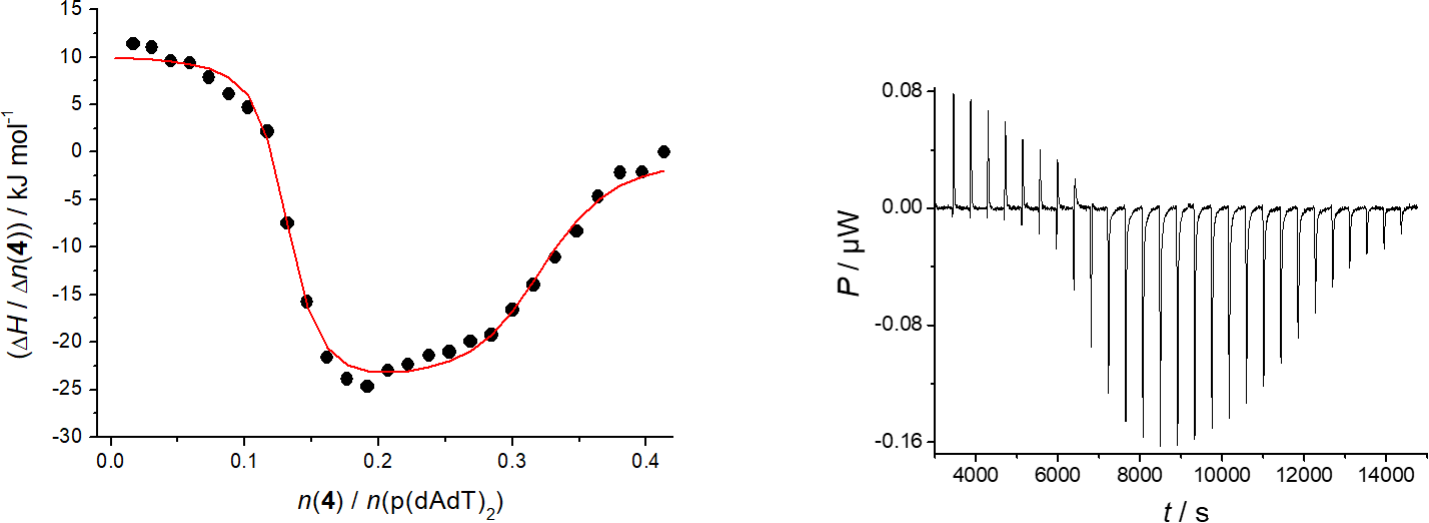
ITC experiments of poly(dAdT)–poly(dAdT) (*c* = 5.0 × 10^−5^ M) titrated with compound **4**. Dots represent experimental data and calculated fit for model “two set of sites” (red line). Inset: original titration data, done at pH 5.0 (Na cacodylate buffer, *I* = 0.05 M).

Intriguingly, the addition of compound **4** to ds-DNAs showed two pronounced and different processes (Figure 5 and Figures S23 and S24 in Supporting Information File 1). Processing of the titration data yielded binding constants log *K*_a1_ (Table 3) similar to those determined by UV–vis titrations (Table 2), whereby an excellent agreement of the values of ratio *n* ≈ 0.2 should be stressed. For poly(A)–poly(U) only one binding process was observed, pointing out that aggregation at high excess of compound **4** (*r* > 0.3) noted in CD experiment (Figure 3b) does not contribute significantly to the binding enthalpy.

**Table 3:** Data parameters observed during nonlinear regression for ITC titration of ct-DNA, poly(A)–poly(U), p(dAdT)_2_, and p(dGdC)_2_ with compound **4** with the model “two set of sites*”* (two binding modes).

Polynucleotide	*n*_1_^a^	log *K*_a1_	Δ_r_*H*_1_/kJ mol^−1^	Δ_r_*S*_1_/J K^−1^ mol^−1^	*T*Δ_r_*S*_1_/kJ mol^−1^	Δ_r_*G*_1_/kJ mol^−1^

ct-DNA	0.15	6.8	−21.6	58.0	17.3	−38.9
p(dAdT)_2_	0.19	6.7	−25.5	43.2	12.9	−38.4
p(dGdC)_2_	0.18	6.9	−12.8	89.7	26.7	−39.5

(poly(rA)–poly(rU)^b^	0.14	6.9	−12.4	91.2	27.2	−39.6

	*n*_2_^a^	log *β*_a_^c^	Δ_r_*H*_2_/kJ mol^−1^	Δ_r_*S*_2_/J K^−1^ mol^−1^	*T*Δ_r_*S*_2_/kJ mol^−1^	Δ_r_*G*_2_/kJ mol^−1^

ct-DNA	0.12	8.4	4.3	174.0	51.9	−47.6
p(dAdT)_2_	0.12	8.9	10.2	206.0	61.4	−51.2
p(dGdC)_2_	0.28	9.5	−6.7	160.0	47.7	−54.4

^a^Ratio *n* = [**4**]/[polynucleotide]. ^b^Fitted to single set of binding sites. ^c^log *β*_a_* =* log *K*_a1_ + log *K*_a2_.

The analysis of the binding parameters collected in Table 3 at excess of DNA/RNA over compound **4** (*n* < 0.3, binding to primary binding site) revealed a large negative reaction enthalpy change and a large positive reaction entropy change, indicating favourable enthalpic (exothermic) and entropic contribution to the reaction Gibbs free energy change. This means that the reaction is both enthalpically and entropically driven. However, secondary binding site (*n* > 0.4; excess of **4** over DNA primarily binding site) is thermodynamically expressed only for alternating DNAs, characterised by positive (favourable) binding entropies (*T*∆*S* term) and mostly positive or weakly negative enthalpies (Table 3), revealing an entropically driven binding*.* Positive reaction enthalpy changes (endothermic reaction) suggest the disruptions of the energetically favourable non-covalent interactions, hydrogen bonds, and van der Waals interactions formed between the DNA/RNA and solvent, and between the compound **4** and solvent. The large positive entropy changes suggest solvent release upon binding, which makes a favourable contribution to the reaction Gibbs free energy change. Moreover, at these conditions (*n* > 0.6) also strong new ICD bands are visible, pointing out that binding of surplus molecules of **4** is not random but well organised along the chiral double helix.

**Figure 6:**
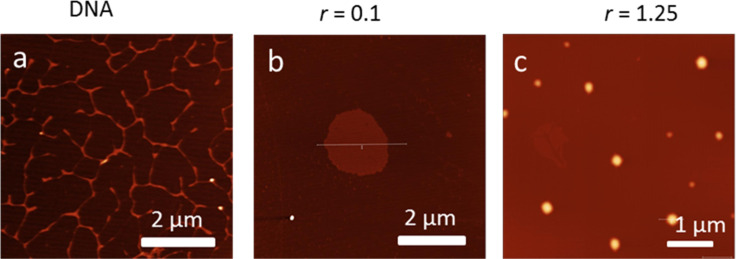
AFM image of a) ct-DNA showing a fibre-like structure with a length of several micrometres; b) upon mixing with compound **4** at ratio *r*_[_**_4_**_]/[ct-DNA]_ = 0.1 and 1.25 showing a spherical polyplex structure with diameter of 2 μm, or c) 500 nm. Done at pH 5, Na cacodylate buffer, *I* = 0.05 M.

Furthermore, such highly positively charged systems, which efficiently wrap around DNA, usually also cause DNA condensation, as we have shown previously for some GCP analogues [16]. Thus, we performed AFM (Figure 6) and DLS experiments (Figure S25, Supporting Information File 1) at conditions of excess of **4** over ct-DNA.

The AFM images clearly showed that the elongated structure of the free ct-DNA (Figure 6a) with a height of 2 nm (see Supporting Information File 1, Figure S26 for the height profile) and a length of a few micrometres, after the addition of compound **4** (*r*_[_**_4_**_]/[DNA]_ = 0.1) formed disk-like aggregates with a diameter of 2 μm and a height of approximately 1 nm (Figure 6b, height profile shown in Figure S26, Supporting Information File 1). During such early condensation at a low ratio (large excess of DNA over dye), the original fibre-like structure of DNA started to form into ragged, dye-non-saturated species that may lead to lowering the height of the complex. At even higher *r* values (*r* = 1.25, excess of dye over DNA) more compact condensed DNA structures were observed with a diameter of 500 nm and a height of 10 nm (Figure 6c, see Supporting Information File 1, Figure S26 for height profile), so called polyplexes.

For the estimation of the condensed particle size at more biologically relevant conditions (in aqueous solution) we used DLS. Pure ct-DNA gave rise to particles with an average diameter of 44 nm (Supporting Information File 1, Figure S25). The addition of compound **4** to the DNA led to the formation of condensed aggregates with a diameter increase of 60 and 115 nm at ratios of *r* = 0.1 and 0.5, respectively. Higher ratios resulted in even larger particle size, which eventually ended in precipitation.

## Conclusion

We designed and synthesized a novel naphtalene diimide analogue equipped at imide positions with two guanidinio-carbonyl-pyrrole (GCP) pendant arms. The GCP was added for their known pH-dependent control of binding to DNA/RNA [15–22]. Indeed, the novel conjugate **4** showed significantly stronger affinity and thermal stabilisation effects at pH 5 than at pH 7, which corroborated by CD results was attributed to **4** threading intercalation into ds-DNAs at pH 5, while compound **4** at neutral conditions (pH 7) switched to the DNA minor groove binding. As often observed for threading intercalators, compound **4** was selective toward GC-DNA. Intriguingly, compound **4** was at both pH values bound to the ds-RNA major groove, most likely due to the particular deep and narrow shape of the groove (Table S2 in Supporting Information File 1), still showing a higher affinity and thermal denaturation effect at pH 5 due to GCP protonation.

At crowding conditions (excess of **4** over DNA/RNA binding sites), the studied compound showed pronounced aggregation, which could be attributed to the electrostatic interactions of the highly positively charged molecules along the negatively charged DNA/RNA backbone aided by the strong hydrophobic effect of the central NDI core. This effect yielded a marked ds-DNA condensation ability of compound **4**, as was demonstrated by AFM and DLS results.

Since agents that efficiently condense DNA are often employed for cell-transfection purposes, in future prospects of the here presented results we plan to pursue this application as we have shown previously for some GCP analogues [33].

## Experimental

### General

All reagents were purchased from commercial sources (Sigma-Aldrich) and were used as received. Solvents were dried and distilled before use. Millipore water was obtained from a Micropure System from TKA. All reactions were carried out in oven-dried glassware. Lyophilisation was carried out with an Alpha 1-4 2D plus freeze-drying apparatus from Christ. For analytical TLC SiO_2_-coated aluminum foils ALUGRAM SIL G/UV254 from MachereyNagel were used. Reversed-phase column chromatography was performed on an Armen Instrument Spot Flash Liquid Chromatography MPLC with RediSep C-18 Reversed-Phase columns. Microwave-assisted SPPS was carried out using a CEM Discover system. ^1^H and ^13^C NMR spectra were recorded on a DRX 500 MHz Bruker spectrometer at ambient temperature. Chemical shifts (δ) are expressed in parts per million and *J* values in hertz. The following abbreviations were used for peak multiplicities: s, singlet; d, doublet, m, multiplet; br, broad. MALDI–TOF mass spectra were recorded on a Bruker BioTOF III. The UV–vis spectra were recorded on a Varian Cary 100 Bio spectrophotometer and CD spectra on JASCO J815 spectrophotometer at 25 °C using appropriate 1 cm path quartz cuvettes. The determination of pH values was carried out with a pH-Meter 766 Calimatic from Knick. For study of interactions with DNA and RNA, aqueous solutions of compound buffered to pH 7.0 or pH 5.0 (buffer sodium cacodylate, *I* = 0.05 M) were used.

### Synthetic procedures

Synthesis of compound **2** [34]: The mono-Boc-protected ethylenediamine (2 equiv) was added to 1,4,5,8-naphthalenetetracarboxylic dianhydride (**1**, 1 equiv) in dry DMF. The mixture was stirred for 15 h at 90 °C. The crude product was extracted into the organic (chloroform) layer and concentrated under vacuum. The crude product was further purified by column chromatography (1% methanol in chloroform). Yield 80%.

^1^H NMR (300 MHz, CDCl_3_, Figure S27, Supporting Information File 1) δ 8.73 (s, 4H, ArH), 4.90 (br. s, 2H, NH), 4.38 (br. s, 4H, CH2), 3.6–3.54 (m, 4H, CH2), 1.21 (s, 18H, (CH_3_).

Synthesis of **3**: To a solution of Fmoc-Lys(Boc)-OH (2 equiv) and HCTU (2 equiv) in dry DMF, DIPEA (8 equiv) was added and the solution stirred at room temperature for 1 h under an argon atmosphere. The Boc-deprotected compound of **2** (1 equiv) was then added to the solution and the reaction mixture was stirred at room temperature for 24 h under an argon atmosphere. Then, water (100 mL) was added and the yellow precipitate was filtered, and washed with ether. The crude product was further purified by column chromatography (1% methanol in chloroform). Yield 50%.

^1^H NMR (300 MHz, DMSO-*d*_6_, Figure S28, Supporting Information File 1) δ 8.59 (br. s, 4H, NDI), 8.04 (br. s, 2H, NH), 7.87–7.81 (m, 4H, ArH), 7.64–7.58 (m, 4H, ArH), 7.42–7.20 (m, 10H, ArH), 6.90 (br. s, 2H, NH), 4.13–4.03 (m, 10H, CH_2_), 3.75–3.53 (m, 5H, CH_2_), 2.84–2.67 (m, 5H, CH_2_), 1.35 (s, 18H, Boc ), 1.28–1.21 (m, 6H, aliphatic proton).

Synthesis of **4**: To a solution of pyrrole carboxylic acid (**A**, 2 equiv), HCTU (2 equiv) in dry DMF and DIPEA (8 equiv) were added, and the solution was stirred at room temperature for 1 h under an argon atmosphere. The corresponding Boc-deprotected amine from **3** (1 equiv) was then added to the solution and the reaction mixture was stirred at room temperature for 24 h under an argon atmosphere. Then, water (100 mL) was added and the yellow precipitate was filtered, and washed with ether. After Boc and Fmoc deprotection by using TFA and piperidine, respectively, crude compound **4** was obtained. The crude product was further purified by column chromatography (1% methanol in chloroform). Yield 50%.

Mp 230–235 °C; ^1^H NMR (300 MHz, DMSO-*d*_6_, Figure S29, Supporting Information File 1) δ 12.16 (br. s, 2H, NH), 11.79 (br. s, 1H, NH), 8.64 (s, 4H, NDI-ArH), 8.51–8.46 (m, 9H, NH), 8.15 (br. s, 3H, NH), 7.36 (br. s, 2H, pyrrole CH), 6.77 (br. s, 2H, pyrrole CH), 4.10–3.88 (m, 5H, CH_2_), 1.64–1.02 (m, 15H, aliphatic proton); ^13^C NMR (75 MHz, DMSO-*d*_6_, Figure S30, Supporting Information File 1) δ 171.1, 162.8, 159.8, 158.7, 155.3, 132.2, 130.2, 126, 125.9, 120, 118, 116.1, 114.1, 113.7, 38.6, 26.6, 22.4; MALDI–TOF–HRMS, Figure S31, Supporting Information File 1, (*m*/*z*): [M + H]^+^ calcd for C_44_H_52_N_16_O_10_, 965.4052; found, 965.4163;

### Interactions with DNA and RNA

Polynucleotides were purchased as noted: poly(rA)–poly(rU), poly(dA)–poly(dT), poly(dAdT)–poly(dAdT), and poly(dGdC)–poly(dGdC) (Sigma) and calf thymus (ct)-DNA (Aldrich). Polynucleotides were dissolved in sodium cacodylate buffer, *I* = 0.05 M, pH 7.0. Calf thymus (ct)-DNA was additionally sonicated and ﬁltered through a 0.45 µm ﬁlter [35–36]. The polynucleotide concentration was determined spectroscopically [36] as the concentration of phosphates. Spectrophotometric titrations were performed at pH 7.0 or pH 5.0 (*I* = 0.05 M, buffer sodium cacodylate) by adding portions of the polynucleotide solution into the solution of the studied compound for UV–vis; CD experiments were done by adding portions of the compound stock solution into the solution of the polynucleotide. Titration data were processed by Scatchard equation [28]. Values for *K*_a_ and *n* given in Table 1 all have satisfactory correlation coefficients (>0.999). Thermal melting curves for DNA, RNA, and their complexes with the studied compound were determined as previously described [37] by following the absorption change at 260 nm as a function of temperature. The absorbance of the ligands was subtracted from every curve and the absorbance scale was normalized. *T*_m_ values are the midpoints of the transition curves determined from the maximum of the first derivative and checked graphically by the tangent method [36]. The Δ*T*_m_ values were calculated by subtracting *T*_m_ of the free nucleic acid from *T*_m_ of the complex. Every Δ*T*_m_ value reported herein was the average of at least two measurements. The error in Δ*T*_m_ is ±0.5 °C.

Isothermal titration calorimetry (ITC) experiments were performed on a MicroCal VP-ITC microcalorimeter (MicroCal, Inc., Northampton, MA, USA). Origin 7.0 software, supplied by the manufacturer, was used for data analysis. During the titration of ct-DNA, d: poly(rA)–poly(rU), poly(dAdT)–poly(dAdT), and poly(dGdC)–poly(dGdC) with the compound **4**, one aliquot of 2 μL and 27 aliquots of 10 μL of **4** (*c* = 1.0 × 10^−4^ M) were injected from rotating syringe (307 rpm) into the isothermal cell, equilibrated at 25.0 °C, containing 1.4406 mL of polynucleotide (*c* = 3.0–5.0 × 10^−5^ M). The spacing between each injection was 420 s. The initial delay before the first injection was 3000 s in all experiments. All solutions used for ITC experiments were degassed prior to use under vacuum (0.64 bar, 10 min).

### AFM experimental

In a similar manner as described in [16], the samples were prepared by diluting a DNA stock solution (6 mM) in sodium cacodylate buffer (*I* = 0.05 M, pH 5.0) with water and for compound **4** (4 mM stock solution) at various molar ratios of compound **4** to DNA. For each measurement, 10 μL of the mixed solution were deposited onto a freshly cleaned mica surface (Plano GmbH). The sample was dried by means of a spin-coater (1 min at 20 rps and 2 min at 100 rps). For the AFM imaging operating in the tapping mode (scan rate 5 μm s^−1^) with N-type silicon cantilevers (AC-160TS, Olympus) a NanoDrive controller with an Innova scanning probe microscope (Veeco, Germany, Mannheim) was used. The analysis of the AFM images was carried out by use of the Gwyddion (version 2.19) software.

### DLS experimental

All measurements were carried out using a ZetasizerNano ZS from Malvern equipped with a 4 mW He-Ne laser (633 nm wavelength) at a fixed detector angle of 173° with an avalanche photodiode detector in sodium cacodylate buffer (*I* = 0.05 M, pH 5.0) at 25 °C in UV-transparent microcuvettes (1 cm) equipped with a stopper. Mixtures of DNA (50 μM) and compound **4** (5–100 μM) were prepared and filtered prior to measurement through 0.20 μm nylon filters. The autocorrelation functions of the backscattered light fluctuations were analysed with the DTS 6.20 software from Malvern providing the hydrodynamic diameter (Z-average), polydispersity, and size distribution (NNLS analysis).

## Supporting Information

File 1 Spectrophotometric characterisation in solution, NMR and HRMS data, additional experimental data on interactions with DNA/RNA.
