# Supplementary material for: Naphthalene diimide bis-guanidinio-carbonyl-pyrrole as a pH-switchable threading DNA intercalator
Source: Beilstein J Org Chem. 2020 Sep 8;16:2201–11. doi: 10.3762/bjoc.16.185 (PMC7492691; doi:10.3762/bjoc.16.185)
Supplement: File 1 — Spectrophotometric characterisation in solution, NMR and HRMS data, additional experimental data on interactions with DNA/RNA. [file Beilstein_J_Org_Chem-16-2201-s001.pdf]

BEILSTEIN JOURNAL OF ORGANIC CHEMISTRY

Supporting Information

for

Naphthalene diimide bis-guanidinio-carbonyl-pyrrole as a pH-switchable threading DNA intercalator

Poulami Jana, Filip Šupljika, Carsten Schmuck and Ivo Piantanida

Beilstein J. Org. Chem. **2020,** *16,* 2201–2211. doi:10.3762/bjoc.16.185

Spectrophotometric characterisation in solution, NMR and HRMS data, additional experimental data on interactions with DNA/RNA

License and Terms: This is a supporting information file under the terms of the Creative Commons Attribution License (https://creativecommons.org/licenses/by/4.0). Please note that the reuse, redistribution and reproduction in particular requires that the authors and source are credited.
The license is subject to the *Beilstein Journal of Organic Chemistry* terms and conditions: (https://www.beilstein-journals.org/bjoc)

Table of contents

Spectrophotometric characterisation in solution S2

Study of interactions with DNA/RNA S3

NMR, HRMS and HPLC data S17

References S21

Spectrophotometric characterisation in solution

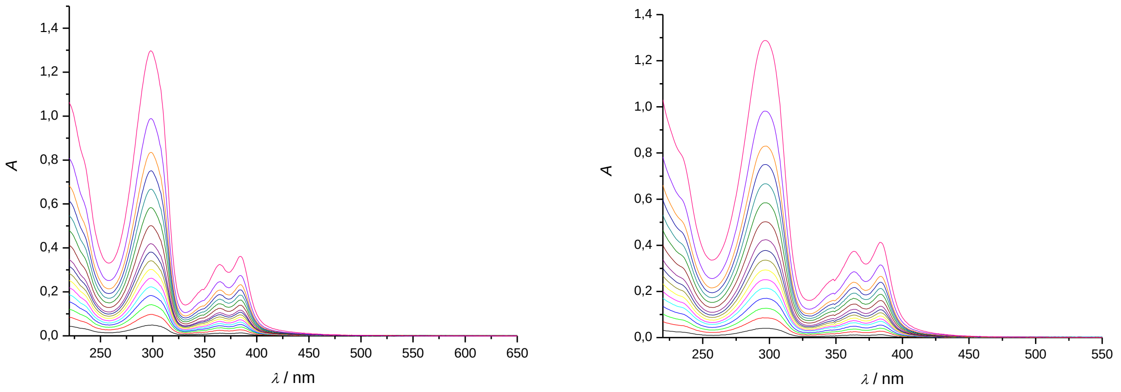

Figure S1: UV–vis calibration spectra of compound **4** (c(**4**) = 1 × 10^{-6} to 3 × 10^{-5} mol dm^{-3}) in 50 mM sodium cacodylate buffer at: LEFT pH 7 ; RIGHT: pH 5.

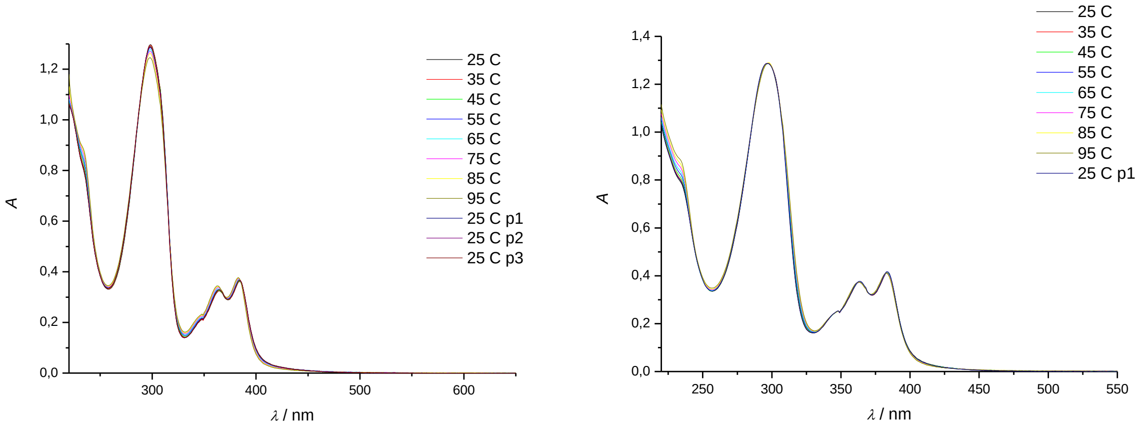

Figure S2: Changes of **4** (c(**4**) = 3 × 10^{-5} mol dm^{-3}) UV–vis spectrum at different temperatures (25–95 °C) in 50 mM sodium cacodylate buffer. LEFT: pH 7; RIGHT: pH 5.

Table S1. Molar absorption coefficients (ε) of compound **4** in sodium cacodylate buffer at pH 7 and pH 5.

pH 7	ε(298 nm)/mmol^{-1} cm^{2}	ε(384 nm)/mmol^{-1} cm^{2}	ε(364 nm)/mmol^{-1} cm^{2}
	41535.4	11564.2	10346.9
pH 5	ε(297 nm)/mmol^{-1} cm^{2}	ε(383 nm)/mmol^{-1} cm^{2}	ε(364 nm)/mmol^{-1} cm^{2}
	42035.1	13442.9	12176.6

Study of interactions with DNA/RNA

Polynucleotides were purchased as noted: poly(rA)–poly(rU), poly(dA)–poly(dT), poly(dAdT)–poly(dAdT), and poly(dGdC)–poly(dGdC) (Sigma) and calf thymus (*ct*)-DNA (Aldrich) and dissolved in sodium cacodylate buffer, *I* = 0.05 M, pH 7.0. The ct-DNA was additionally sonicated and filtered through a 0.45 mm filter to obtain mostly short (ca. 100 basepairs) rod-like B-helical DNA fragments [1]. The polynucleotide concentration was determined spectroscopically [2] as the concentration of phosphates (corresponds to *c*(nucleobase)).

Table S2: Groove widths and depths for selected nucleic acid conformations [3,4].

Structure type	Groove width [Å]		Groove depth [Å]	
	major	minor	major	minor
[a] poly rA – poly rU	3.8	10.9	13.5	2.8
[b] poly dA – poly dT	11.4	3.3	7.5	7.9
[c] poly dGdC – poly dGdC	13.5	9.5	10.0	7.2
[c] poly dAdT – poly dAdT	11.2	6.3	8.5	7.5

[a] A-helical structure (e.g. A-DNA)

[b] C-helical structure (e.g. C-DNA)

[c] B- helical structure (e.g. B-DNA)

1.1 . <u>UV–vis spectrophotometric studies</u>

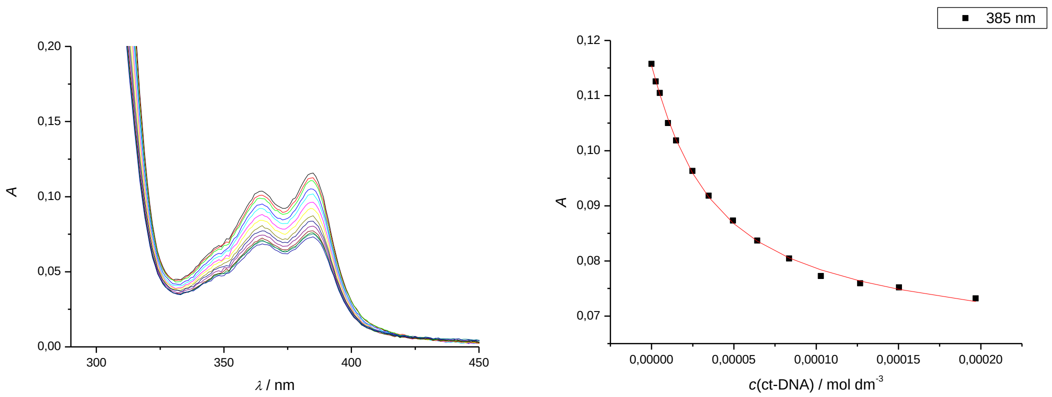

Figure S3: UV–vis spectra and data analysis according to Scatchard equation at the wavelength 385 nm of the interaction of **4** with the ct-DNA in 50 mM sodium cacodylate buffer, pH 7.0.

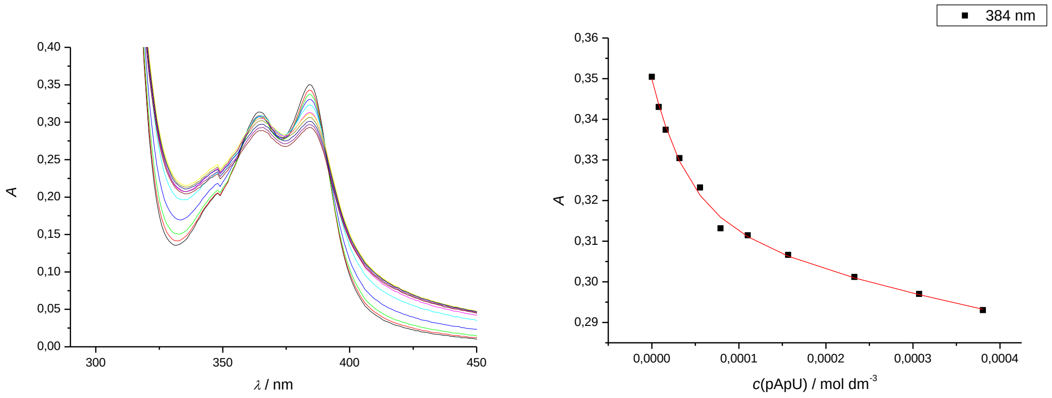

Figure S4: UV–vis spectra and data analysis according to Scatchard equation at the wavelength 384 nm of the interaction of **4** with the poly A - poly U in 50 mM sodium cacodylate buffer, pH 7.0.

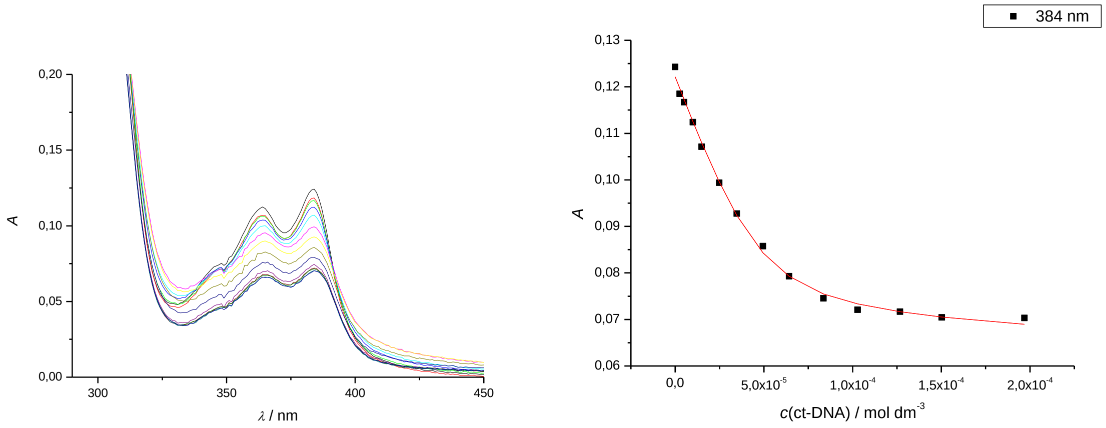

Figure S5: UV–vis spectra and data analysis according to Scatchard equation at the wavelength 384 nm of the interaction of **4** with the ct-DNA in 50 mM sodium cacodylate buffer, pH 5.0.

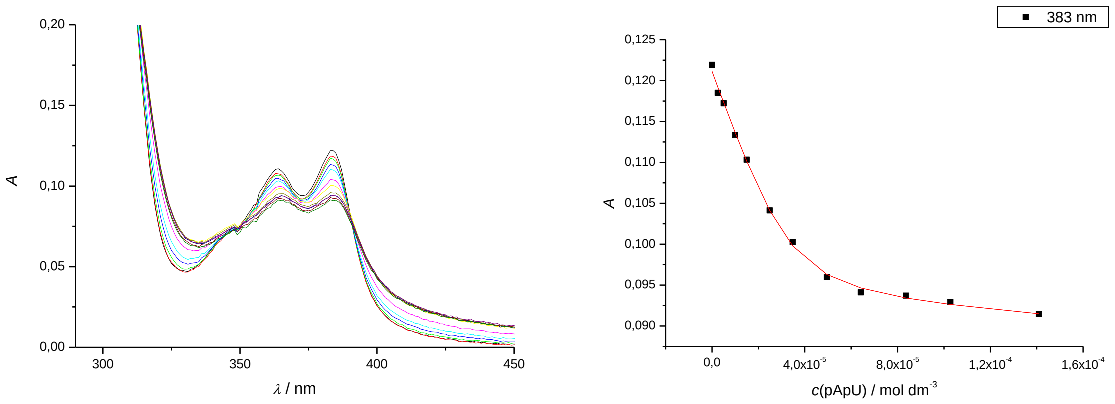

Figure S6: UV–vis spectra and data analysis according to Scatchard equation at the wavelength 383 nm of the interaction of **4** with the poly A - poly U in 50 mM sodium cacodylate buffer, pH 5.0.

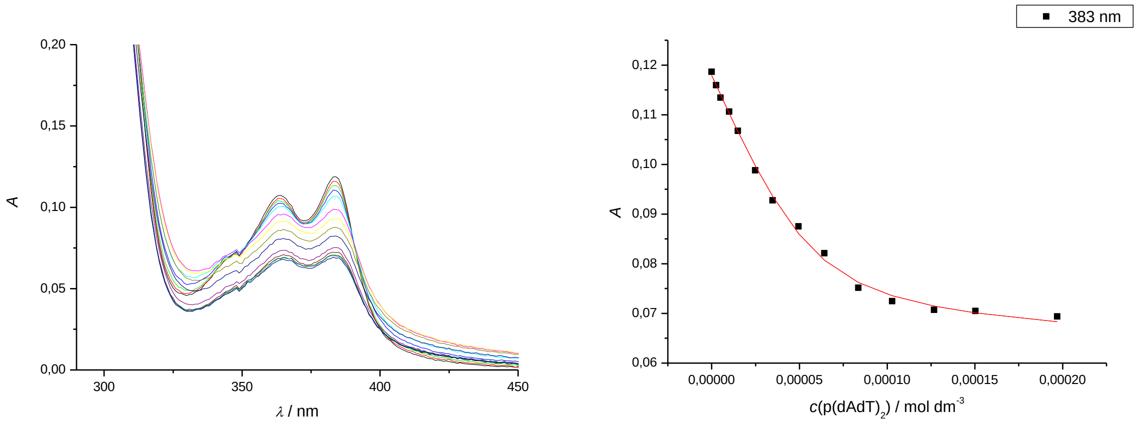

Figure S7: UV–vis spectra and data analysis according to Scatchard equation at the wavelength 383 nm of the interaction of **4** with the $p(dAdT)_2$ in 50 mM sodium cacodylate buffer, pH 5.0.

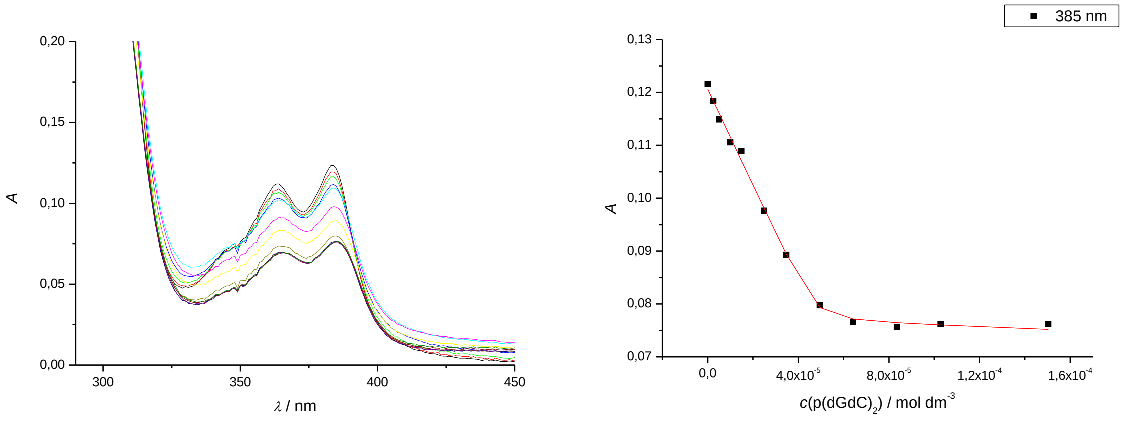

Figure S8: UV–vis spectra and data analysis according to Scatchard equation at the wavelength 385 nm of the interaction of **4** with the p(dGdC)$_2$ in 50 mM sodium cacodylate buffer, pH 5.0

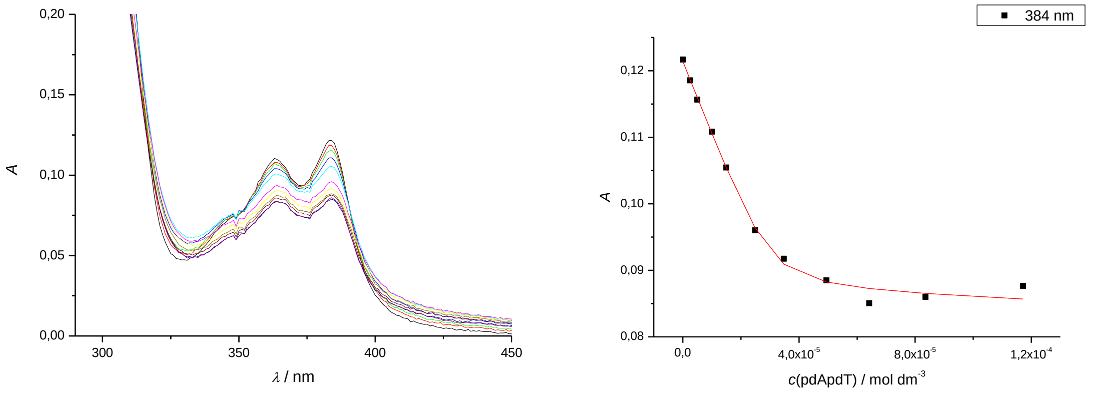

Figure S9: UV–vis spectra and data analysis according to Scatchard equation at the wavelength 384 nm of the interaction of **4** with the pdApdT in 50 mM sodium cacodylate buffer, pH 5.0.

Thermal denaturation experiments:

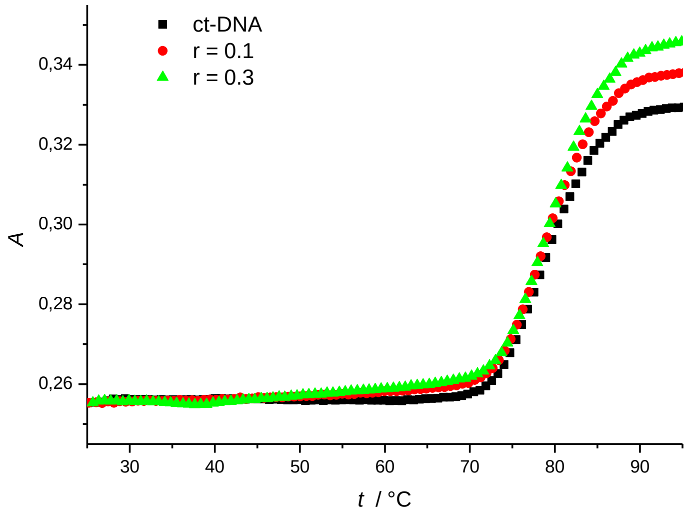

Figure S10: Thermal melting experiments of ct-DNA (▪), *n*(**4**) / *n*(ct-DNA) = 0.1 (●) and *n*(**4**) / *n*(ct-DNA) = 0.3 (▲) in 50 mM sodium cacodylate buffer, pH 7.0.

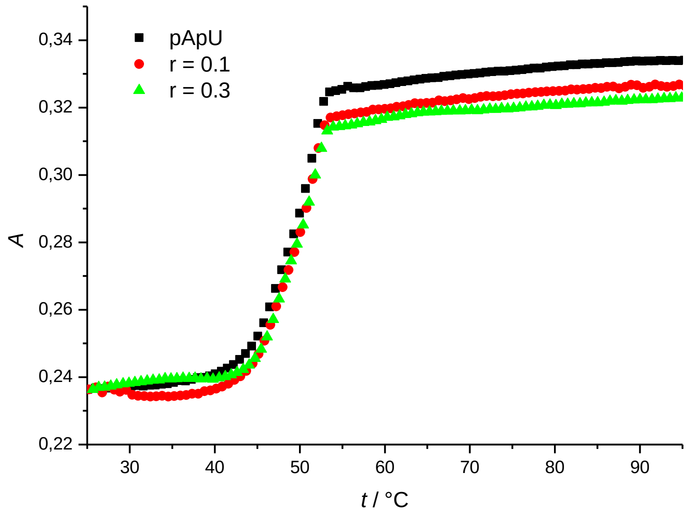

Figure S11: Thermal melting experiments of poly A - poly U (▪), *n*(**4**) / *n*(poly A - poly U) = 0.1 (●) and *n*(**4**) / *n*(poly A - poly U) = 0.3 (▲) in 50 mM sodium cacodylate buffer, pH 7.0.

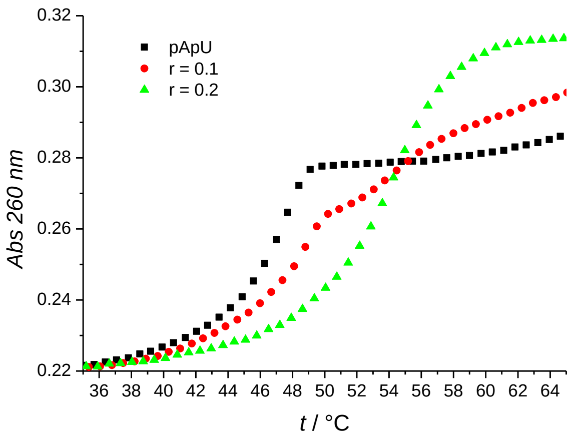

Figure S12: Thermal denaturation of poly rA – poly rU ($c = 2 \times 10^{-5}$ M) upon addition of **4** at various ratios $r_{[\mathbf{4}] / [\text{polynucleotide}]}$. Done at pH 5 (buffer sodium cacodylate, I = 0.05 M).

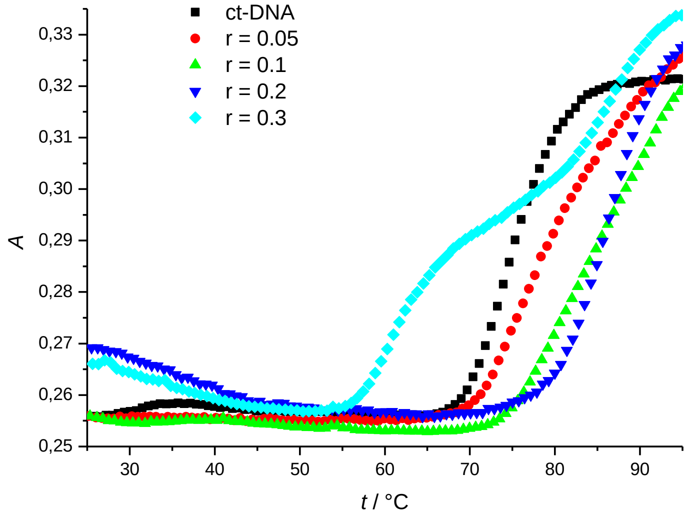

Figure S13: Thermal melting experiments of ct-DNA (▪), n(**4**) / n(ct-DNA) = 0.05 (●), n(**4**) / n(ct-DNA) = 0.1 (▲), n(**4**) / n(ct-DNA) = 0.2 (▼) and n(**4**) / n(ct-DNA) = 0.3 (♦) in 50 mM sodium cacodylate buffer, pH 5.0.

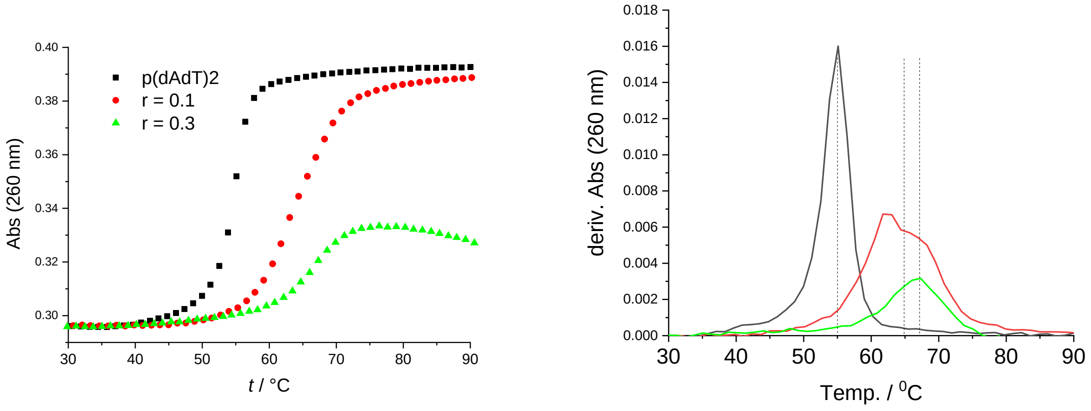

Figure S14: Thermal denaturation experiments of poly (dAdT)$_2$ (c(poly (dAdT)$_2$) = 2.5 $\times$ 10^{-5} M, r $_{[\mathbf{4}]\ /\ [\mathrm{DNA}]}$ = 0.1 ; 0.3) at pH 5.0 (sodium cacodylate buffer, I = 0.05 M).

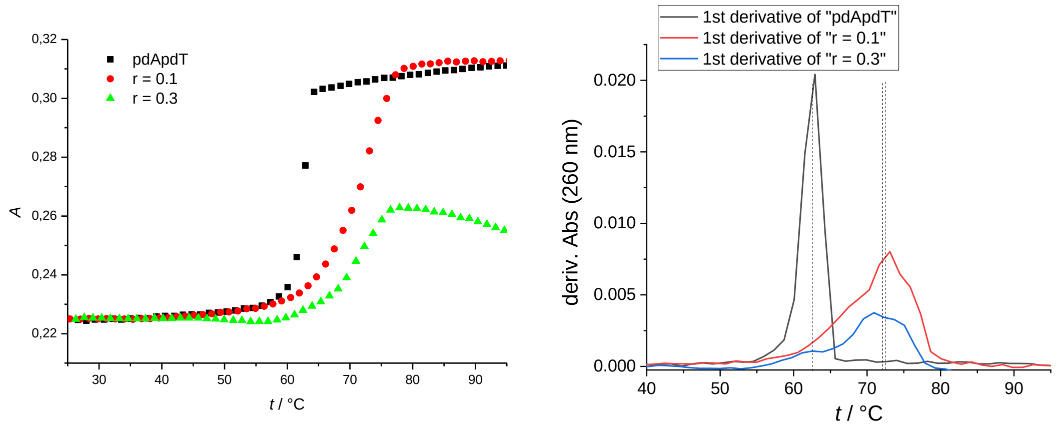

Figure S15: Thermal denaturation experiments of poly dA-poly dT (c(p(poly dA-poly dT) = 2.5 $\times$ 10^{-5} M, r $_{[\mathbf{4}]\ /\ [\mathrm{DNA}]}$ = 0.1 ; 0.3) at pH 5.0 (sodium cacodylate buffer, I = 0.05 M).

1.3. CD measurements

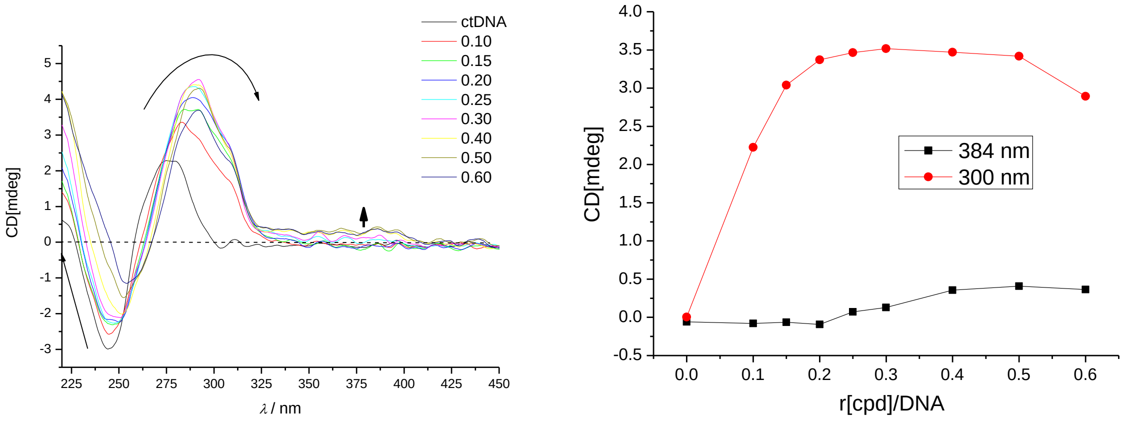

Figure S16: CD titration of ct-DNA ($c = 3 \times 10^{-5}$ mol dm^{-3}) with **4** at different molar ratios r = [4] / [ct-DNA] in 50 mM sodium cacodylate buffer, pH 5.0.

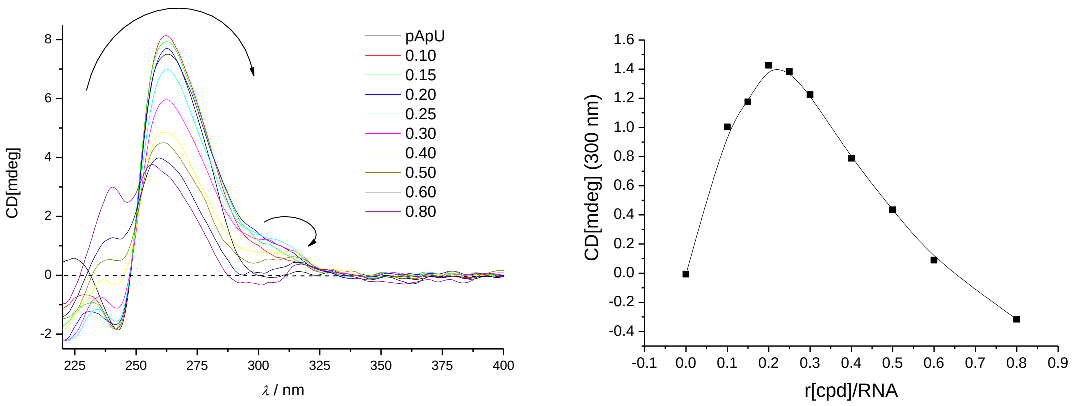

Figure S17: CD titration of poly A - poly U ($c = 3 \times 10^{-5}$ mol dm^{-3}) with **4** at different molar ratios r = [4] / [poly A - poly U] in 50 mM sodium cacodylate buffer, pH 5.0.

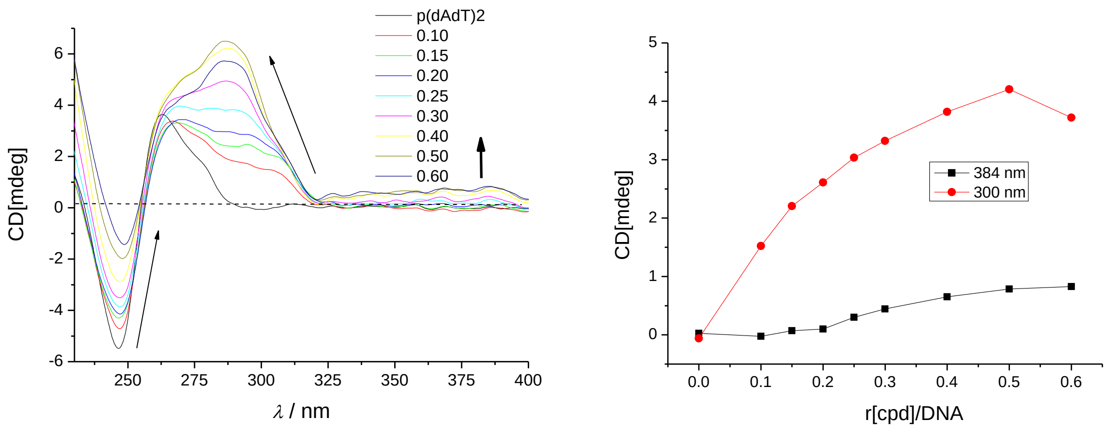

Figure S18: CD titration of p(dAdT)$_2$ (c = 3 × 10^{-5} mol dm^{-3}) with **4** at different molar ratios r = [4] / [p(dAdT)$_2$] in 50 mM sodium cacodylate buffer, pH 5.0.

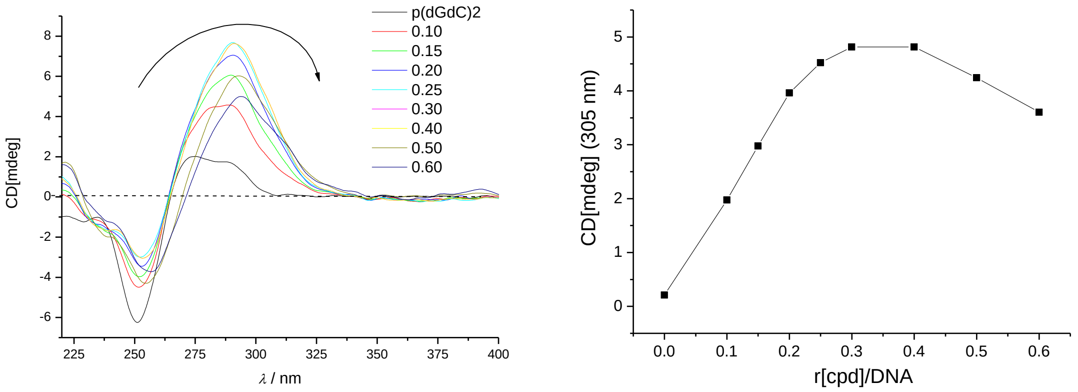

Figure S19: CD titration of p(dGdC)$_2$ (c = 3 × 10^{-5} mol dm^{-3}) with **4** at different molar ratios r = [4] / [p(dGdC)$_2$] in 50 mM sodium cacodylate buffer, pH 5.0.

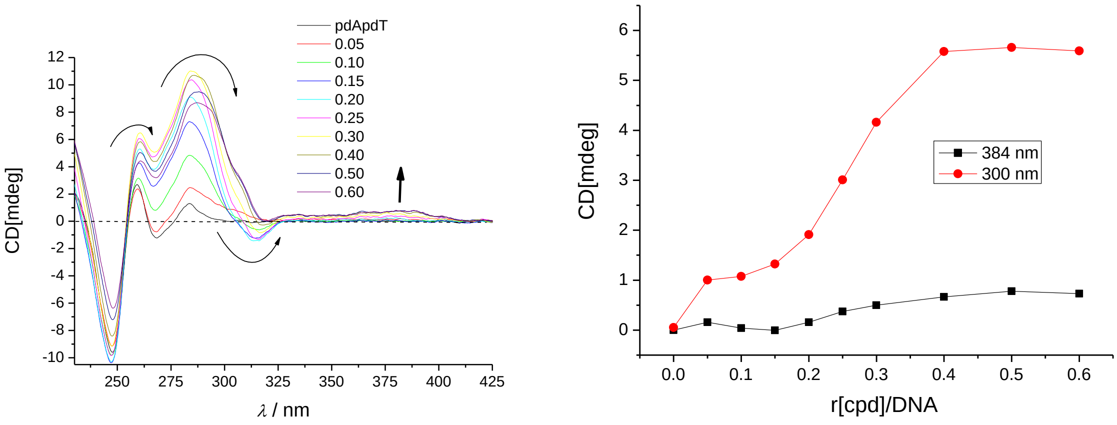

Figure S20: CD titration of pdApdT (c = 3 × 10^{-5} mol dm^{-3}) with **4** at different molar ratios r = [4] / [pdApdT] in 50 mM sodium cacodylate buffer, pH 5.0.

ITC Results:

ITC titration profiles of polynucleotides (ct-DNA, pApU, p(dAdT)$_2$, p(dGdC)$_2$, pdApdT) with compound **4**. Each of the heat burst curves in the figures correspond to a single injection (Figures S21–S24, top panel). The areas under these heat burst curves were determined by integration to yield the associated injection heats, which are plotted against the molar ratio of polynucleotide/ligand. The resulting values were fitted to a one-site or two-sites binding models by the nonlinear least square method. The data points reflect the experimental injection heats while the solid lines represent the calculated fit of the data (Figures S21–S24, curves in the lower panel).

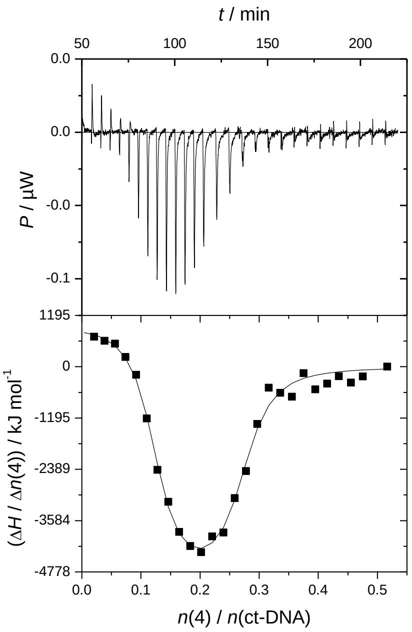

Figure S21: Final figure for ITC titration of ct-DNA with **4** in 50 mM sodium cacodylate buffer, pH 5.

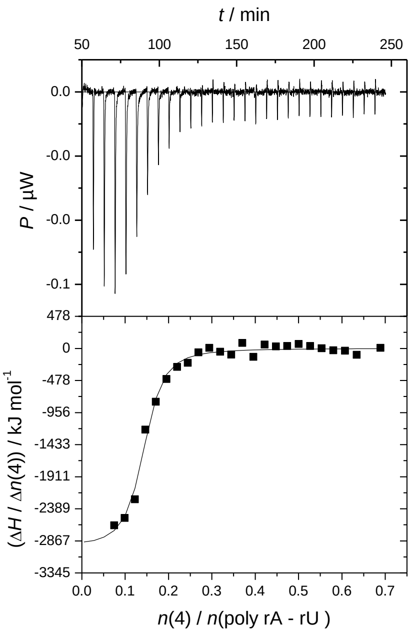

Figure S22: Final figure for ITC titration of poly rA - rU with **4** in 50 mM sodium cacodylate buffer, pH 5.

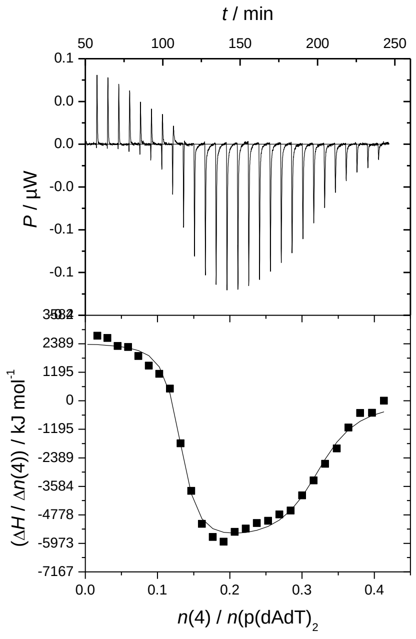

Figure S23: Final figure for ITC titration of $p(dAdT)_2$ with **4** in 50 mM sodium cacodylate buffer, pH 5.

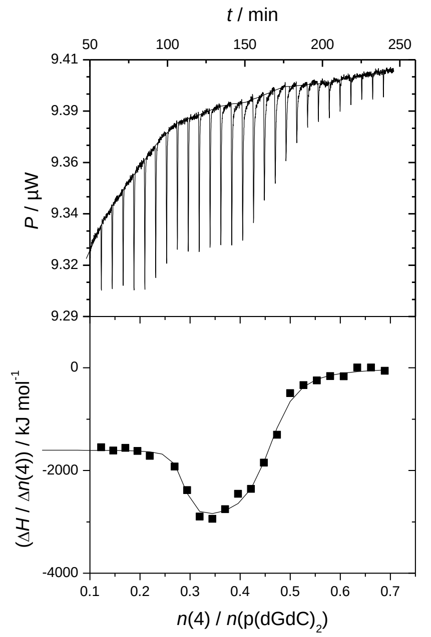

Figure S24: Final figure for ITC titration of p(dGdC)$_2$ with **4** in 50 mM sodium cacodylate buffer, pH 5. The fit is only estimation because of the unstable baseline.

DLS experiments:

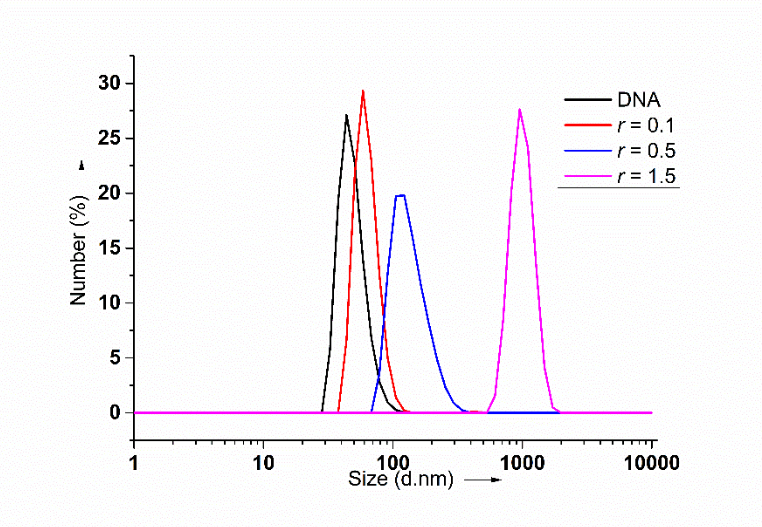

Figure S25: Number size distribution of complex between DNA and **4** obtained from DLS measurements at pH 5, *r* = [**4**/ DNA]. DNA (Blackline); **4** + DNA (*r* = 0.1 redline); **4**+ DNA, *r* = 0.5 (blue line); **4**+ DNA, (*r* = 1.5 pink line).

Table S3: DLS size of **4** / DNA complex at various ratios $r_{[\mathbf{4}] / [\text{polynucleotide}]}$.

Condition	**DLS size distribution by number (nm)**
DNA	44
r = 0.1	60
r = 0.5	115
r = 1.5	957

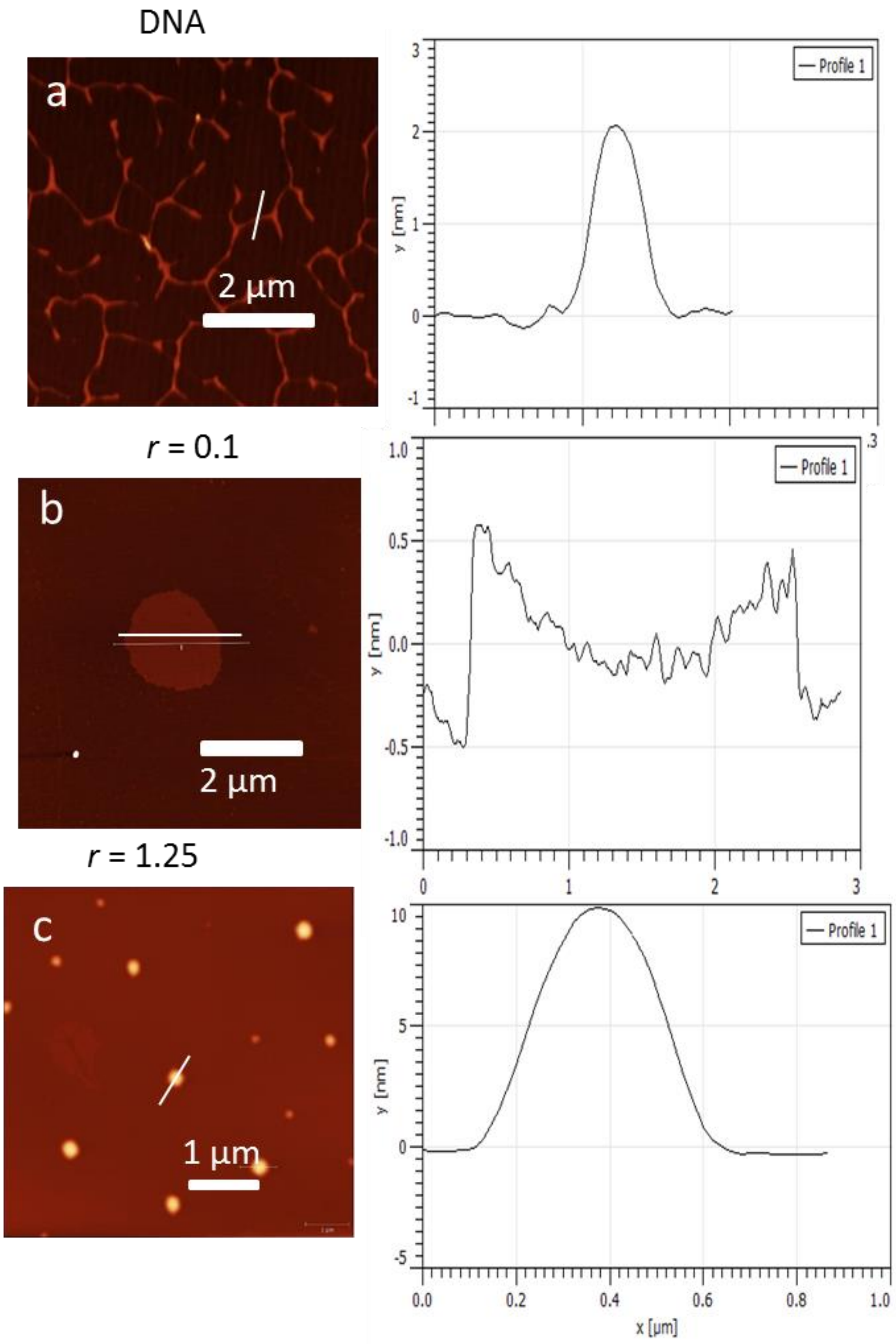

Figure S26: Height profiles for AFM image of ctDNA and **4**/ctDNA complex at various ratios $r_{[\mathbf{4}]/[ctDNA]}$ = 0.1 and 1.25. Done at pH 5, sodium cacodylate buffer, I = 0.05 M.

Synthesis and characterisation:

<u>NMR and HRMS spectra:</u>

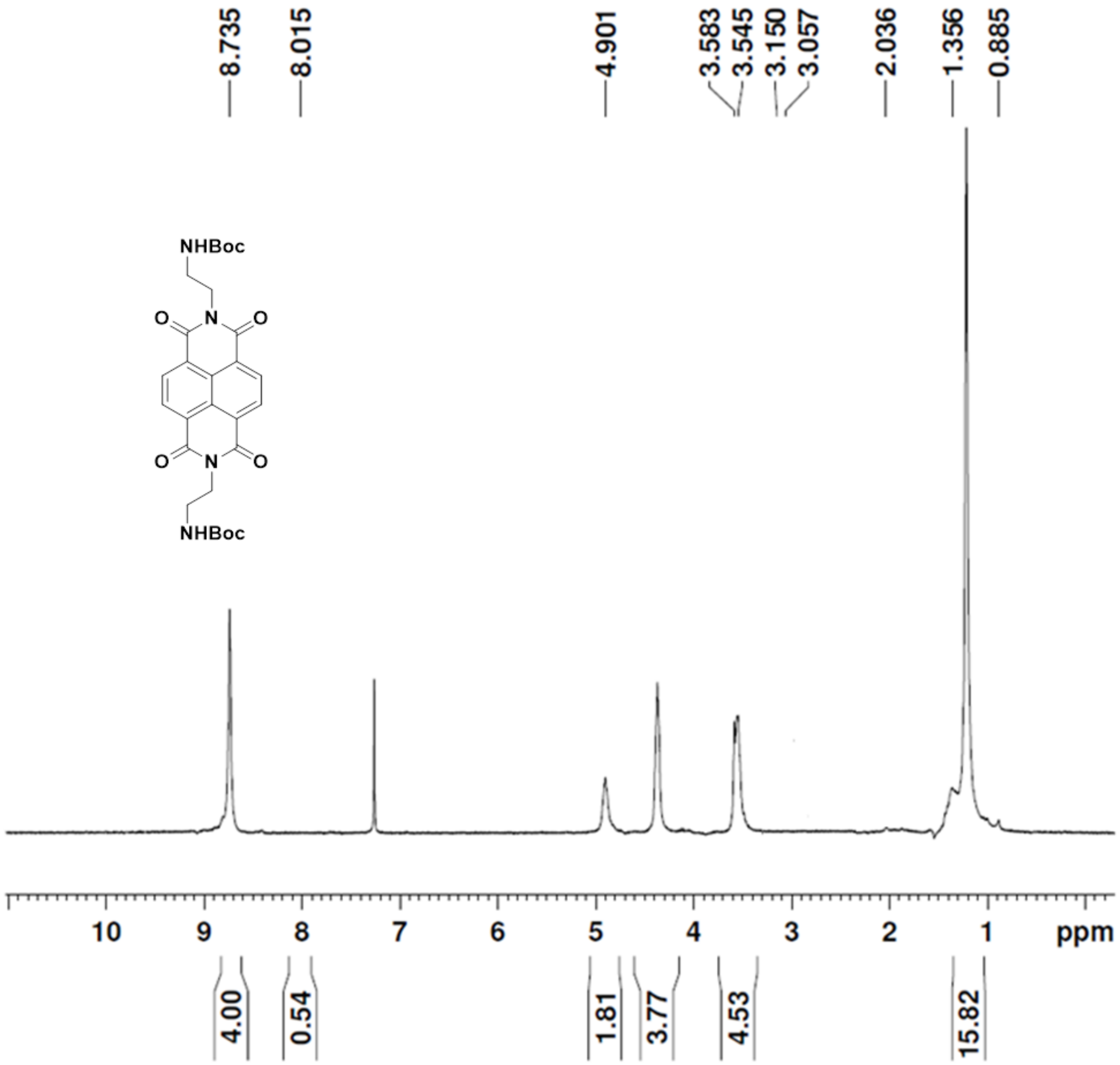

Figure S27: ^{1}H NMR spectrum of compound **2** in $CDCl_3$.

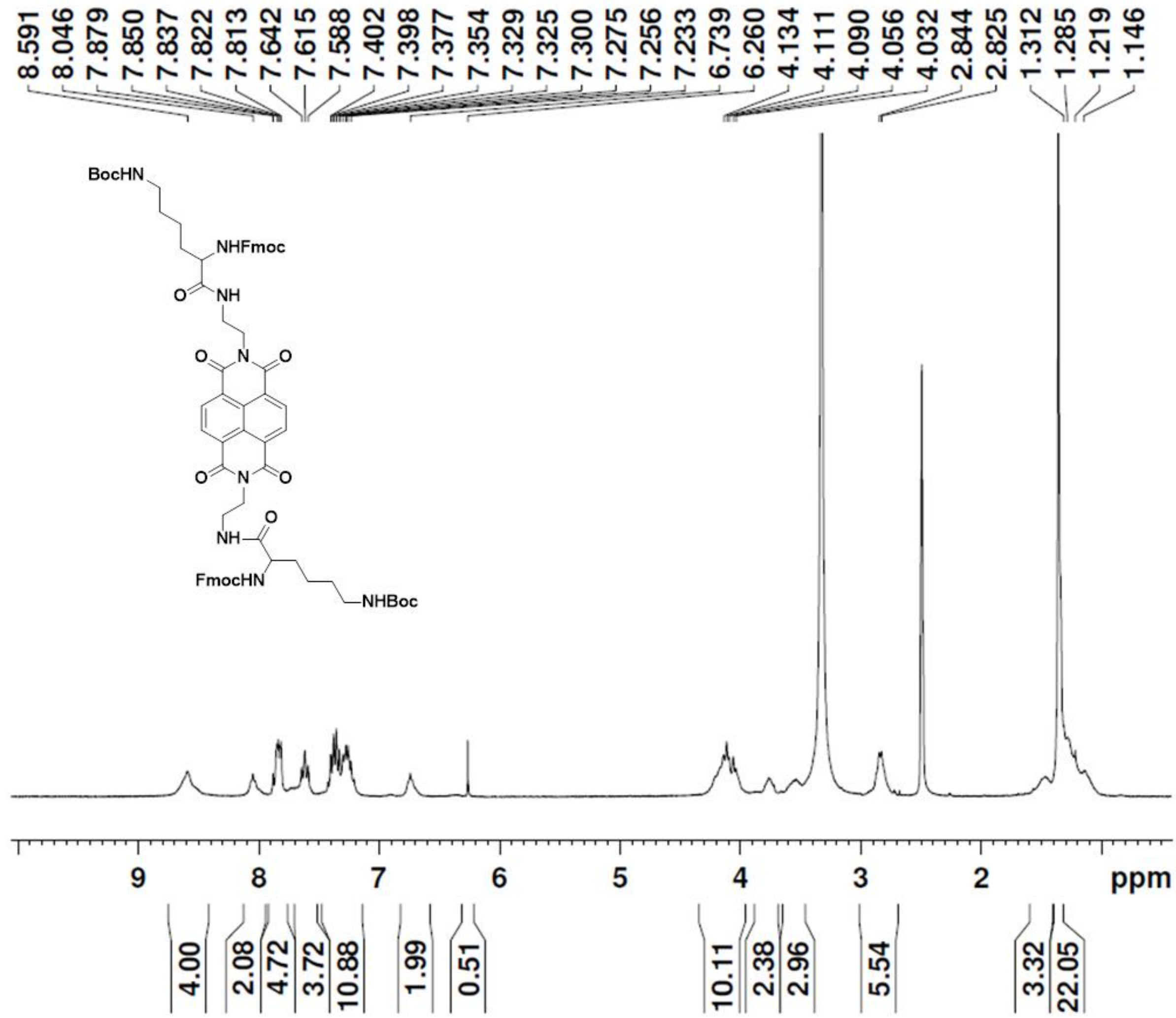

Figure S28: ^{1}H NMR spectrum of compound **3** in DMSO-d_6.

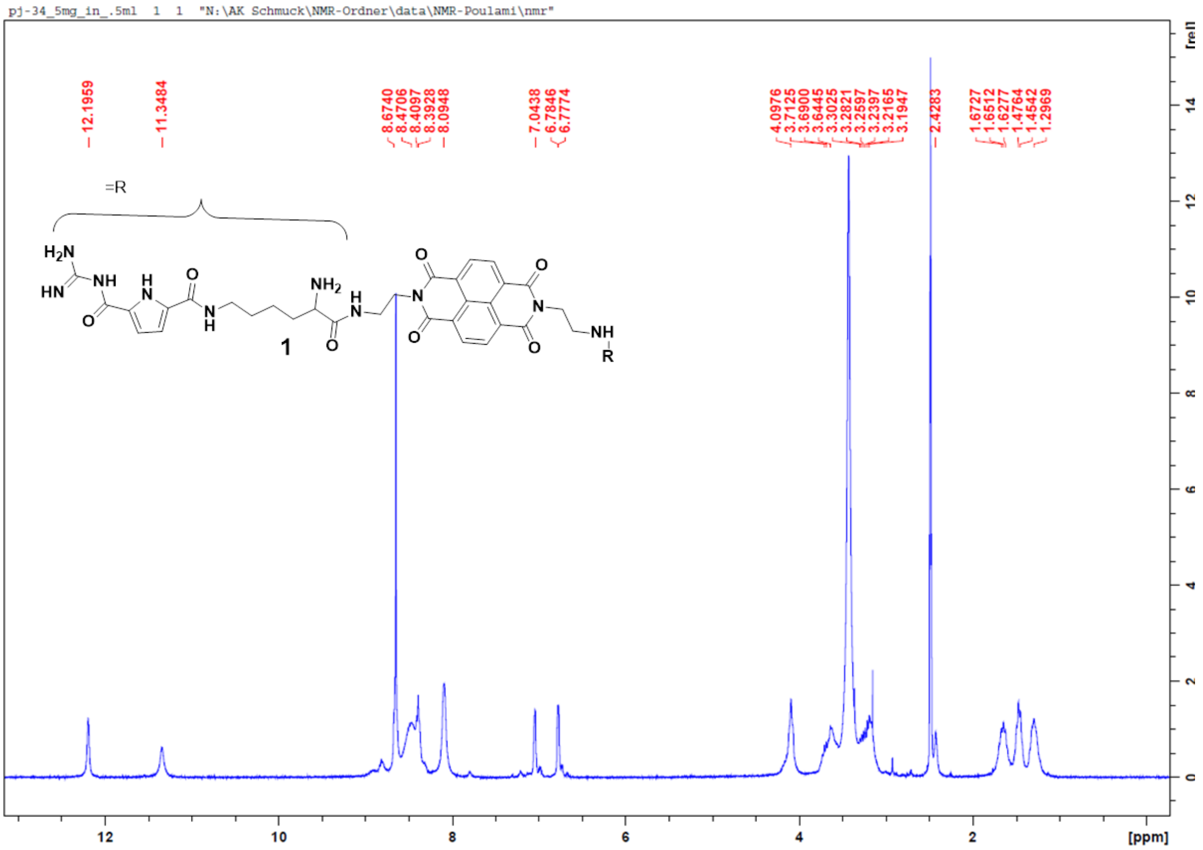

Figure S29: ^{1}H NMR spectrum of compound **4** in DMSO-d_6.

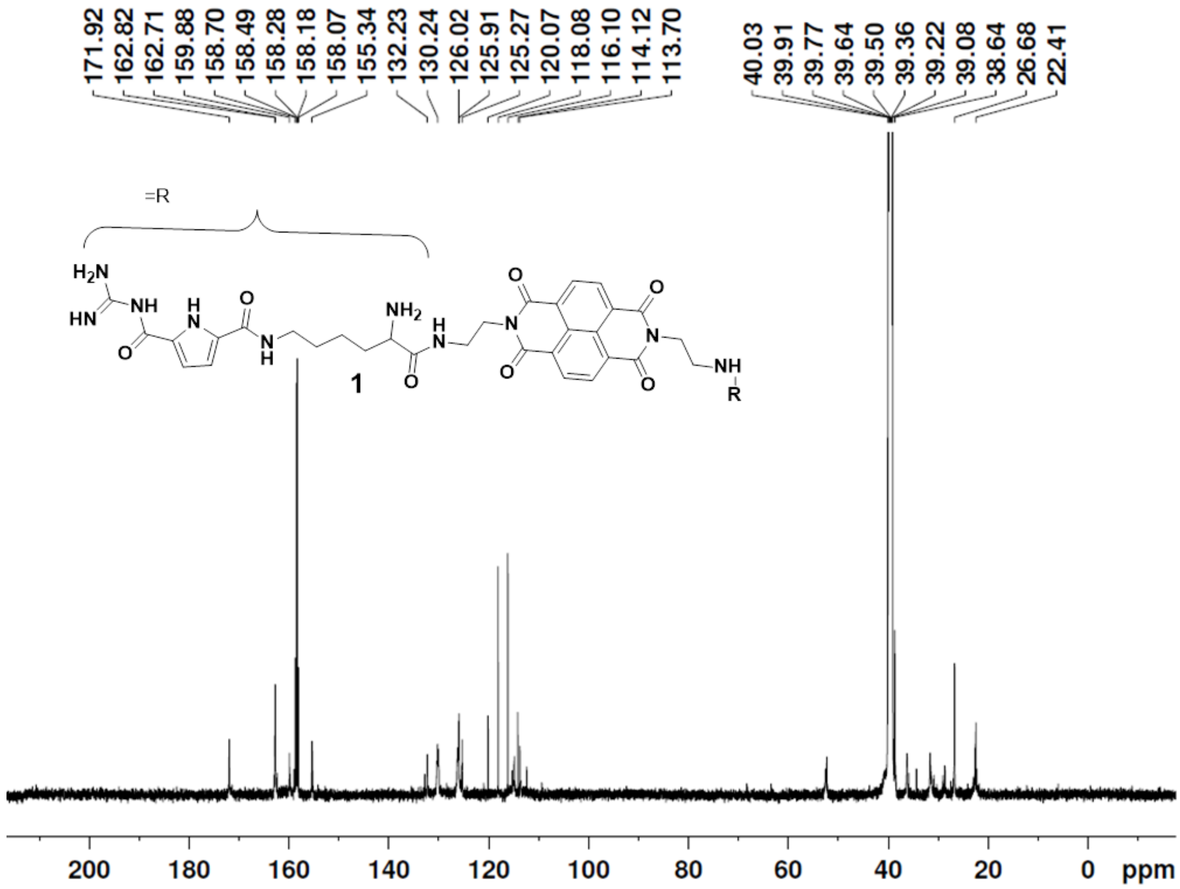

Figure S30. ^{13}C NMR spectrum of compound **4** in DMSO-d_6.

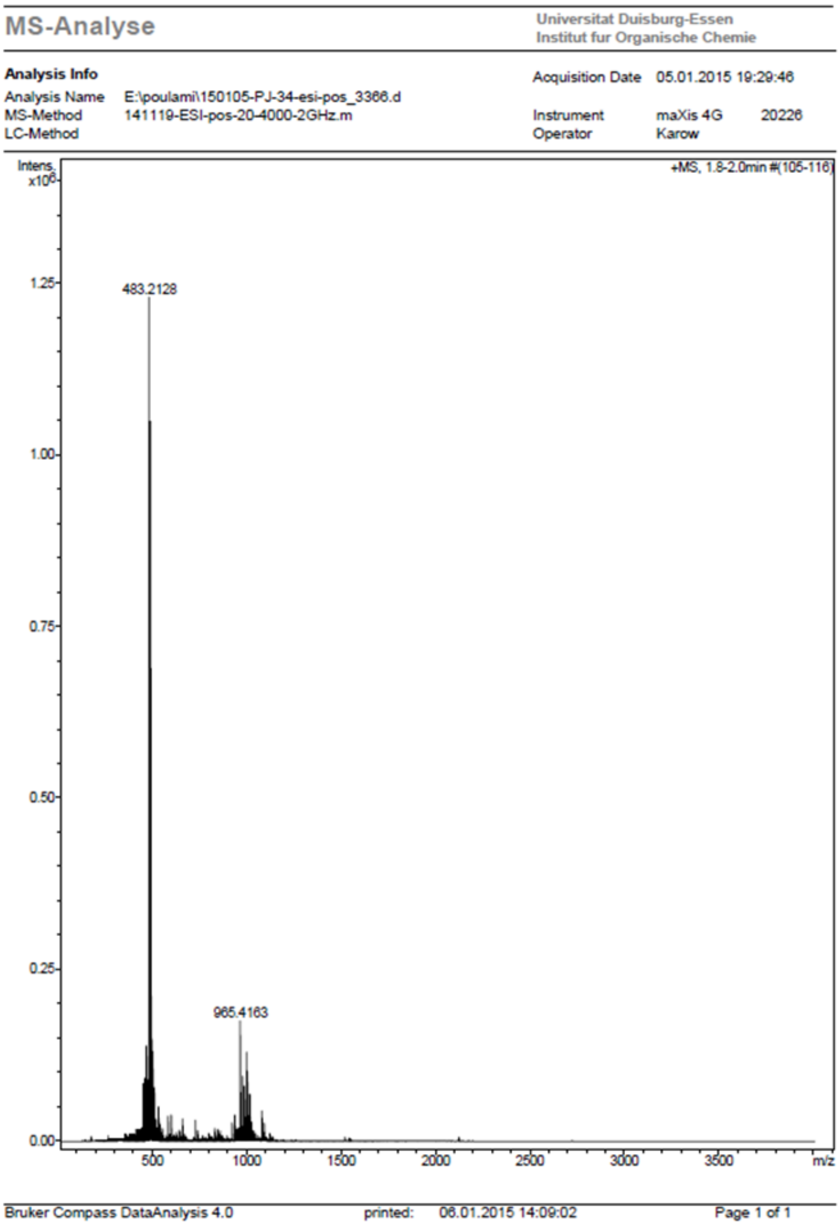

Figure S31: Mass spectrum of compound **4**.

References

[1] Chaires, J. B.; Dattagupta, N.; Crothers, D. M. *Biochemistry* **1982**, *21*, 3933-3940.
[2] Tumir, L. M.; Piantanida, I.; Cindric, I. J.; Hrenar, T.; Meic, Z.; Zinic, M. *J. Phys. Org. Chem.* **2003**, *16*, 891-899.
[3] Saenger, W. *Principles of Nucleic Acid Structure*; Springer-Verlag, New York, 1983.
[4] Cantor, C. R.; Schimmel, P. R. *Biophysical Chemistry* **1980**, *3*, 1109-1181.
